# Neural network-based adaptive global sliding mode MPPT controller design for stand-alone photovoltaic systems

**DOI:** 10.1371/journal.pone.0260480

**Published:** 2022-01-20

**Authors:** Izhar Ul Haq, Qudrat Khan, Safeer Ullah, Shahid Ahmed Khan, Rini Akmeliawati, Mehmood Ashraf Khan, Jamshed Iqbal

**Affiliations:** 1 Department of Electrical and Computer Engineering, COMSATS University, Islamabad, Pakistan; 2 Centre for Advanced Studies in Telecommunications (CAST), COMSATS University, Islamabad, Pakistan; 3 School of Mechanical Engineering, The University of Adelaide, Adelaide, South Australia, Australia; 4 Department of Computer Science and Technology, Faculty of Science and Engineering, University of Hull, Hull, United Kingdom; Huazhong University of Science and Technology, CHINA

## Abstract

The increasing energy demand and the target to reduce environmental pollution make it essential to use efficient and environment-friendly renewable energy systems. One of these systems is the Photovoltaic (PV) system which generates energy subject to variation in environmental conditions such as temperature and solar radiations. In the presence of these variations, it is necessary to extract the maximum power via the maximum power point tracking (MPPT) controller. This paper presents a nonlinear generalized global sliding mode controller (GGSMC) to harvest maximum power from a PV array using a DC-DC buck-boost converter. A feed-forward neural network (FFNN) is used to provide a reference voltage. A GGSMC is designed to track the FFNN generated reference subject to varying temperature and sunlight. The proposed control strategy, along with a modified sliding mode control, eliminates the reaching phase so that the sliding mode exists throughout the time. The system response observes no chattering and harmonic distortions. Finally, the simulation results using MATLAB/Simulink environment demonstrate the effectiveness, accuracy, and rapid tracking of the proposed control strategy. The results are compared with standard results of the nonlinear backstepping controller under abrupt changes in environmental conditions for further validation.

## 1 Introduction

Due to the increased usage of electrical and electronic devices, the power demand is growing every day. The increasing energy demand and environmental pollution worldwide develop fascinations for the introduction of renewable power systems. At the same time, the use of fossil fuels reduces their reserves. Solar energy is one of the most prominent renewable energy sources for power generation. It dominates several other renewable energy sources like wind power, hydro-power, geothermal energy, biofuels, and biomass due to its clean, endless and free nature [1, 2]. At the end of 2017, solar power generation has made a recorded history of adding 98 Giga Watt (GW) to the global installed capacity, making it a total of 402GW [3]. The International Energy Agency (IEA) [4] estimates that by 2050, the photovoltaics (PV) will provide around 11% of global electricity production and would avoid 2.3 Giga ton (Gt) of *CO*_2_ emissions per year.

**Fig 1:**
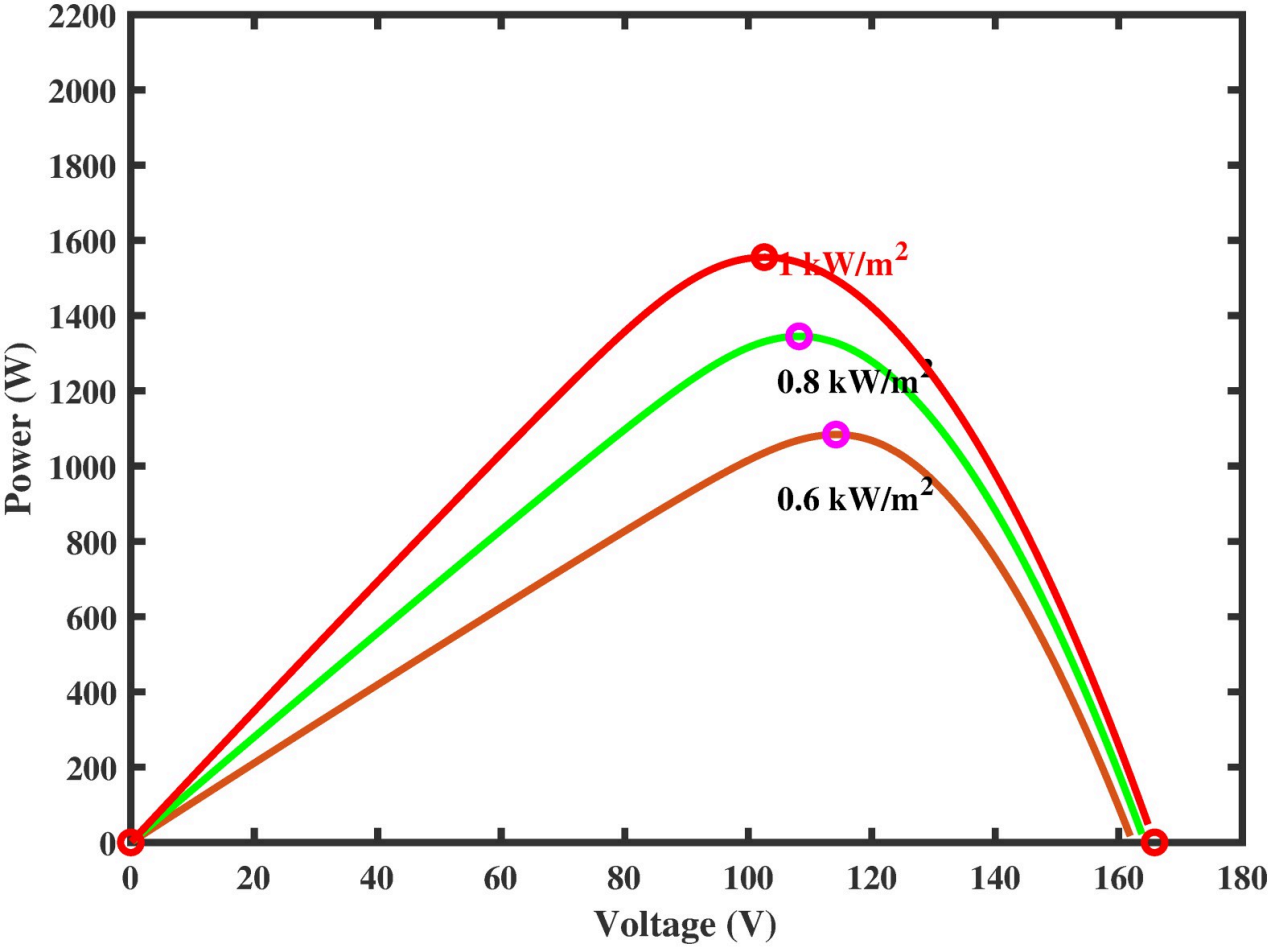
Power characteristic curve under varying irridiance.

**Fig 2:**
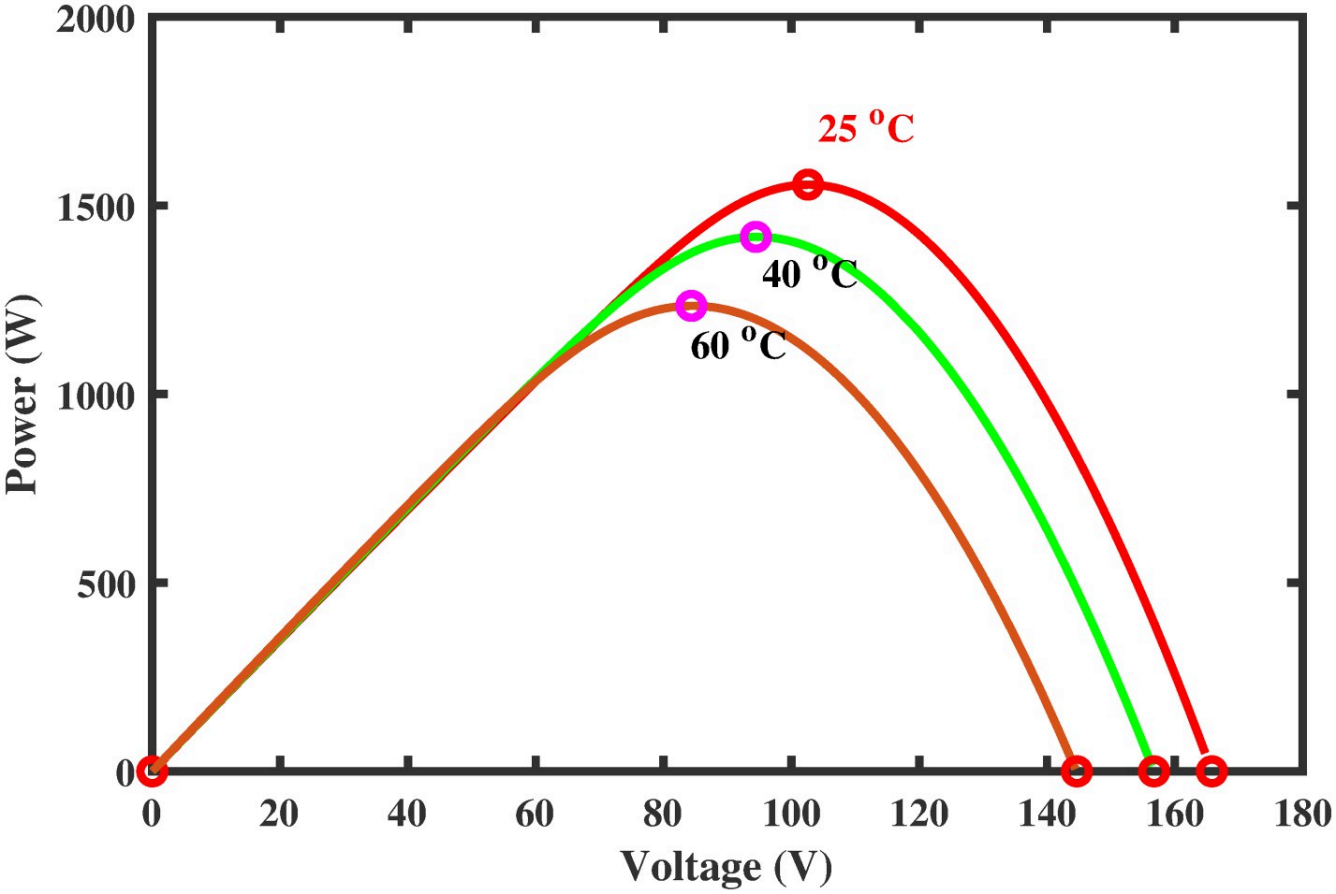
Power characteristic curve under varying temperature.

PV arrays have the advantage of directly converting light energy into electrical energy through semiconductors [5]. However, the main problem is its low efficiency due to varying environmental conditions. To increase the efficiency, they must be operated at the maximum power point (MPP). The operating point of the PV array for maximum power generation is termed as maximum power point and the voltage at which the PV module can produce maximum power is named maximum power voltage (or peak power voltage). MPPT is an essential part of solar power systems; therefore, intensive research work is carried out in this particular area to develop new and more efficient MPPT techniques [6]. The power characteristics, shown in Figs 1 and 2 of photovoltaic cells, are nonlinear that vary with the variations in the environmental conditions [7]. For instance, variation in temperature and irradiance change the voltage produced as well as the generated current by PV module [8]. As a result, the generated power also varies. Such variations make maximum power extraction a complex task.

In the context of maximum power extraction, various techniques are used, which are divided into conventional and soft computing methods. Among the conventional techniques, perturbation and observation (P&O) [9, 10] and incremental conductance (IC) [11] are the most widely used techniques because of their simple structure. In the P&O method, the voltage V and current I from the PV module are measured and the power P is calculated. If the change in power is greater than zero, i.e. (Δ*P* > 0), then it measures the change in voltage. If a positive change occurs in voltage, the duty command ratio is decreased and if a negative change occurs in the voltage, then the duty command ratio is increased. If the change in power is less than zero, i.e. (Δ*P* < 0), then it again measures the change in the voltage. If a change in voltage is positive, the duty command ratio is increased and if the change in voltage is negative, the duty command ratio is decreased. If power remains the same, i.e. (Δ*P* = 0), then it means that MPP is achieved. When the MPP is achieved, voltage oscillates around the MPP rather than being steadily situated on it. This is one of the major weaknesses of this technique. Likewise, as the voltage is tracked occasionally, a large amount of power drops. Similarly, in IC, the maximum power point occurs when Δ*V*/Δ*P* = 0 and Δ*I*/Δ*P* = 0. IC method offers good performance under rapidly changing atmospheric conditions [11]. It is also capable of knowing that the MPP has reached. Consequently, no oscillations occur like those which happens in P&O method [12]. In other words, it is relatively more efficient than P&O method. However, it requires four sensors to perform the computation; thus, it is expensive and requires more computational time, which results in power loss (see for instance; [13, 14]). Although IC is relatively faster than P&O it is still slow and inaccurate in case of faster variations in solar irradiance [15, 16]. Moreover, these conventional techniques have no capability to handle partial shading conditions (PSC), which is a very important issue for PV systems. To address this problem, many MPPT techniques are proposed in the existing literature like dynamic leader based collective intelligence (DLCI), memetic salp swarm algorithm (MSSA), etc., [17–19].

In recent years, due to drawbacks of conventional techniques, researchers have been more attracted by soft computing techniques, which are further classified as bio-inspired methods and artificial intelligence methods [8, 20, 21]. In Bio-inspired techniques, particle swarm optimization (PSO) [22], ant colony optimization (ACO) [23] and genetic algorithms (GA) [24] are commonly used. These proposed controllers under varying weather conditions show fast convergence as compared to conventional methods. The main drawbacks of the Bio-inspired methods are that they need many parameters such as population size, mutation, selection of chromosomes and crossover rate. The estimation of these parameters is itself a complex job. Under varying environmental conditions, all these parameters need to be readjusted; otherwise, one cannot track MPP correctly. In addition, adaptive/robust control algorithms are developed in many control applications. In a robust context, a well-known robust control algorithm, named as sliding mode control (SMC), is designed in comprehensive case studies to ensure the aforementioned control objectives [25, 26]. However, the conventional SMC may experience some high-frequency oscillations that are dangerous in realistic applications [27].

Artificial intelligence-based techniques for the MPPT of PV modules include fuzzy logic (FL) controllers and artificial neural networks (ANN). These techniques have advantages such as working with variable inputs, no need for exact mathematical modeling, self convergence and self-learning capabilities [28]. They have adaptive nature for the nonlinear behavior of the systems. FL MPPT method has an excellent performance as compared to the conventional techniques [29]. The drawback of the FL MPPT method is that the tracking performance and the output efficiency are highly dependent on the engineer’s technical knowledge and the rules-based table. Recently ANN based MPPT techniques are becoming more popular. In [30], an ANN-based MPPT technique is proposed with the advantages of low computation requirement and rapid tracking speed. [31] suggests MPPT technique based on adaptive neuro-fuzzy inference system (ANFIS). [32] have presented the ANN-based MPPT 2-stage method for maximum power point. The ANN MPPT controllers have better performance and effectiveness than the conventional and bio-inspired techniques. Unfortunately, the ANN-based MPPT methods require months for training, large memory size and periodic tuning with time.

This paper proposes a nonlinear GGSMC to extract maximum power from the PV array by tracing a reference voltage. The main contributions of this work are listed below.

1. The proposed control algorithm first generates the reference voltage via FFNN under varying temperatures and irradiances.
2. Then, a non-inverted buck-boost converter, accompanied by an output feedback GGSMC, is used to track the FFNN generated voltage reference.
3. The modified SMC law eliminates the reaching phase so that the sliding mode exists throughout the time. Consequently, MPPT is extracted and the system response observes no high-frequency chattering and harmonic distortions. The overall close loop stability is presented via Lyapunov stability analysis. Furthermore, the simulation results comparison, with existing results of the nonlinear backstepping controller under abrupt changes, demonstrates the effectiveness and faster tracking performance of the proposed SMC over its counterpart.

The rest of the paper is structured as follows. The detail of PV system modeling is given in Section 1 and modeling of the non-inverted buck-boost converter is presented in Section 2. The FFNN used to generate the reference voltage and analysis of the proposed control technique are discussed in Section 3. Simulation results under varying weather conditions are given in Section 4. In Section 5, the performance of the proposed controller is compared with the standard nonlinear backstepping approach. Finally, the conclusion and the future work are presented in Section 6.

## 2 Modelling of PV module

**Fig 3:**
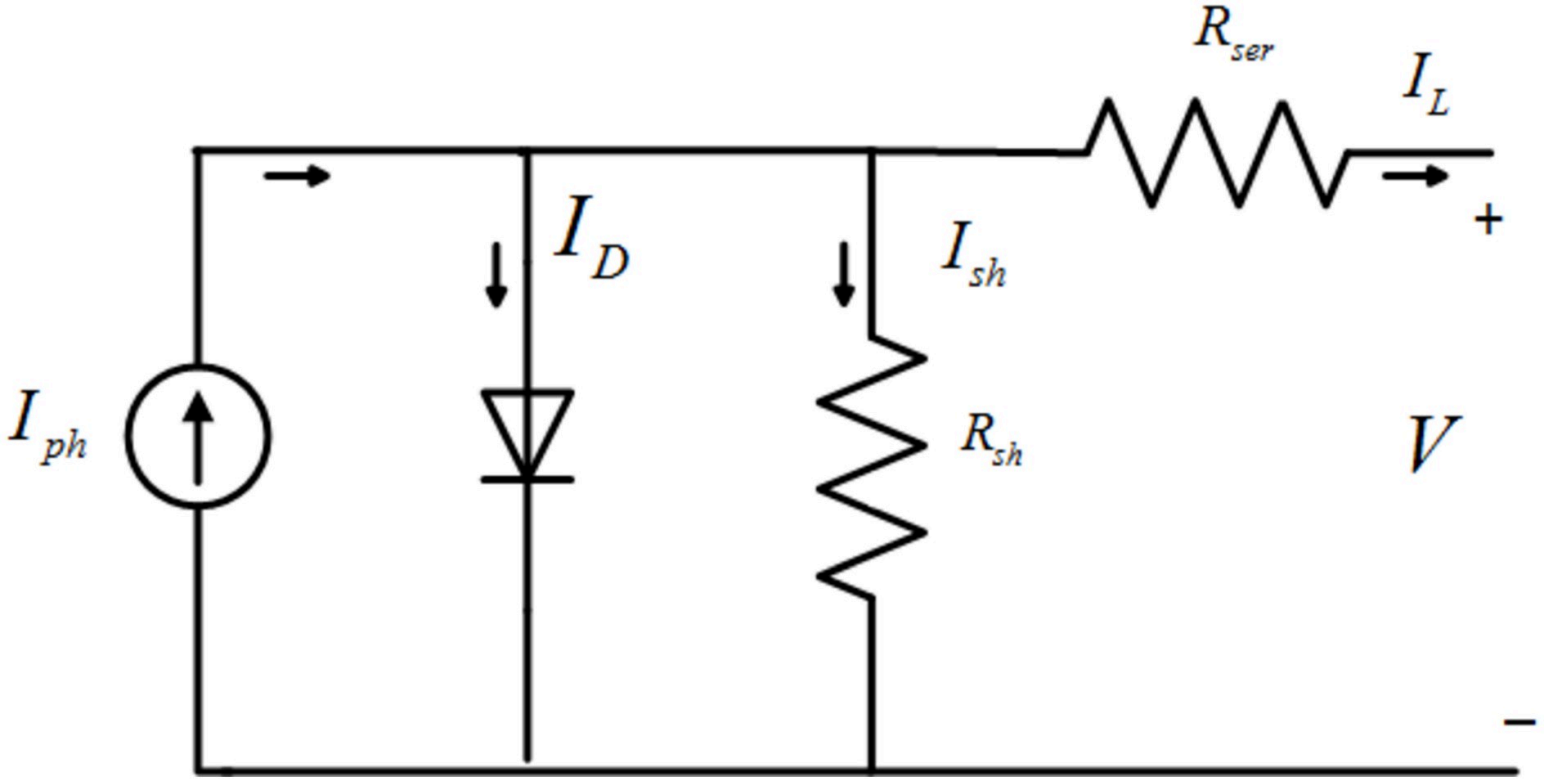
Single diode model of PV module.

A solar cell, the building block of the PV array, is a P-N junction semiconductor. Series and parallel combinations of these solar cells make a PV array. The solar cell is modeled by different equivalent circuits such as single diode model, double diode model and with or without series and shunt resistance [33]. For modeling and simulation purposes, a single diode model is the most commonly used solar cell model. The detailed equivalent single diode model of a PV solar cell [33, 34] is shown in Fig 3. The labeled current equations and output voltage are presented below.

By applying Kirchhoff’s laws, the overall current across the circuit can be calculated as

$$\begin{array}{c} I_{L}=N_{p}I_{ph}-I_{D}-I_{sh} \end{array}$$ (1)

where *I*_*L*_ is the output current of the PV module, *I*_*ph*_ is the photo current which directly depends on the irradiance and temperature, *N*_*p*_ is the parallel connected solar cells, *I*_*D*_ is the current across the diode and *I*_*sh*_ is the current passes through the shunt resistance *R*_*sh*_. The detailed presentation of *I*_*ph*_, *I*_*D*_ and *I*_*sh*_ is given as follow [34]

$$\begin{array}{c} I_{ph}=\left[I_{SCR}+K_{i}\left(T-Tref\right)\frac{S}{S_{ref}}\right] \end{array}$$ (2)

where *I*_*SCR*_ is the cell short circuit current at reference temperature (*T*_*ref*_) and solar irradiance (*S*_*ref*_), which are 25°C and 1000*w*/*m*^2^ respectively, *T* is the cell temperature and *S* is the solar irradiance at current condition and *K*_*i*_ is the short circuit current temperature coefficient.

The diode current is expressed by Shockley’s equation as

$$\begin{array}{c} I_{D}=I_{rs}N_{p}\left(\text{exp}\frac{V_{D}}{nV_{t}}-1\right) \end{array}$$ (3)

where *I*_*rs*_ is the reverse saturation current of the diode, $V_{D}=\frac{V+R_{ser}I_{L}}{N_{s}}$ is the voltage across the diode and $V_{t}=\frac{KT}{q}$ is the thermal voltage while *N*_*s*_ is the number of cells connected in series, *q* = 1.6 × 10^−9^ *C* is the electron charge and *K* = 1.38 × 10^−23^ *J*/*k* is the Boltzmann’s constant

Invoking the detailed values of *V*_*D*_ and *V*_*t*_ in (3), one gets

$$\begin{array}{c} I_{D}=I_{rs}N_{p}\left[\text{exp}\frac{q(V+R_{ser}I_{L})}{nKTN_{s}}\right)] \end{array}$$ (4)

The reverse saturation current *I*_*rs*_ is expressed as

$$\begin{array}{c} I_{rs}=I_{rn}{\left(\frac{T}{T_{ref}}\right)}^{3}\text{exp}\left[\frac{E_{g}}{nK}\left(\frac{1}{T_{ref}}-\frac{1}{T}\right)\right] \end{array}$$ (5)

In this expression, *n* is the diode ideality factor, *I*_*rn*_ is the nominal reverse saturation current at given temperature *T*, *V* is the output voltage of the PV module, and *E*_*g*_ = 1.12*eV* is the bandgap energy of the semiconductor used in the PV cell.

The current across shunt resistance *R*_*sh*_ is given as

$$\begin{array}{c} I_{sh}=\frac{V+R_{ser}I_{L}}{R_{sh}} \end{array}$$ (6)

where *R*_*ser*_ is the series resistance in the PV module, which is produced due to series and parallel joining of the cells of the module and *R*_*sh*_ is the shunt resistance considered due to the leakage current across the P-N junction of the cells of the module.

The values of *R*_*ser*_ = 179.94Ω and *R*_*sh*_ = 3.1694Ω used in MATLAB/Simulink PV module. is used in our simulation. Incorporating (2), (3), and (6) in (1), a complete PV module for this output current can be calculated as

$$\begin{array}{c} \begin{array}{c} I_{L}=N_{p}\left[\left[I_{SCR}+K_{i}\left(T-T_{ref}\right)\frac{S}{1000}\right]-I_{rs}N_{p}\left[\text{exp}\frac{q(V+R_{ser}I_{L})}{nKTN_{s}}\right)\right]-\frac{V+R_{ser}I_{L}}{R_{sh}} \end{array} \end{array}$$ (7)

The temperature coefficient of an open circuit voltage for a PV module *V*_*oct*_ tells the precise relation of open-circuit voltage *V*_*oc*_ with temperature *T* specifying how *V*_*oct*_ changes with changing temperature.

*V*_*oct*_ at variable temperature *T* is expressed as

$$\begin{array}{c} V_{oct}=V_{oc}\left(1+\beta \left(T-25\right)\right) \end{array}$$ (8)

while *β* = −0.36411 is temperature coefficient which means that *V*_*oct*_ decreases at the rate of 0.36411 percent per degree rise in temperature and *V*_*oc*_ is the short circuit current at 25*C*.°

Similarly, the temperature coefficient of short circuit current *I*_*sct*_ also specifies the dependency of short-circuit current *I*_*SC*_ on temperature via the following relation.

$$\begin{array}{c} I_{sct}=I_{SC}\left(1+\alpha \left(T-25\right)\right) \end{array}$$ (9)

while *α* = 0.057006 is temperature coefficient which means that *I*_*sct*_ increases at the rate of 0.057006 percent per degree rise in temperature and *I*_*SC*_ is the short circuit current at 25*C*.°

## 3 Modeling of buck boost converter

**Fig 4:**
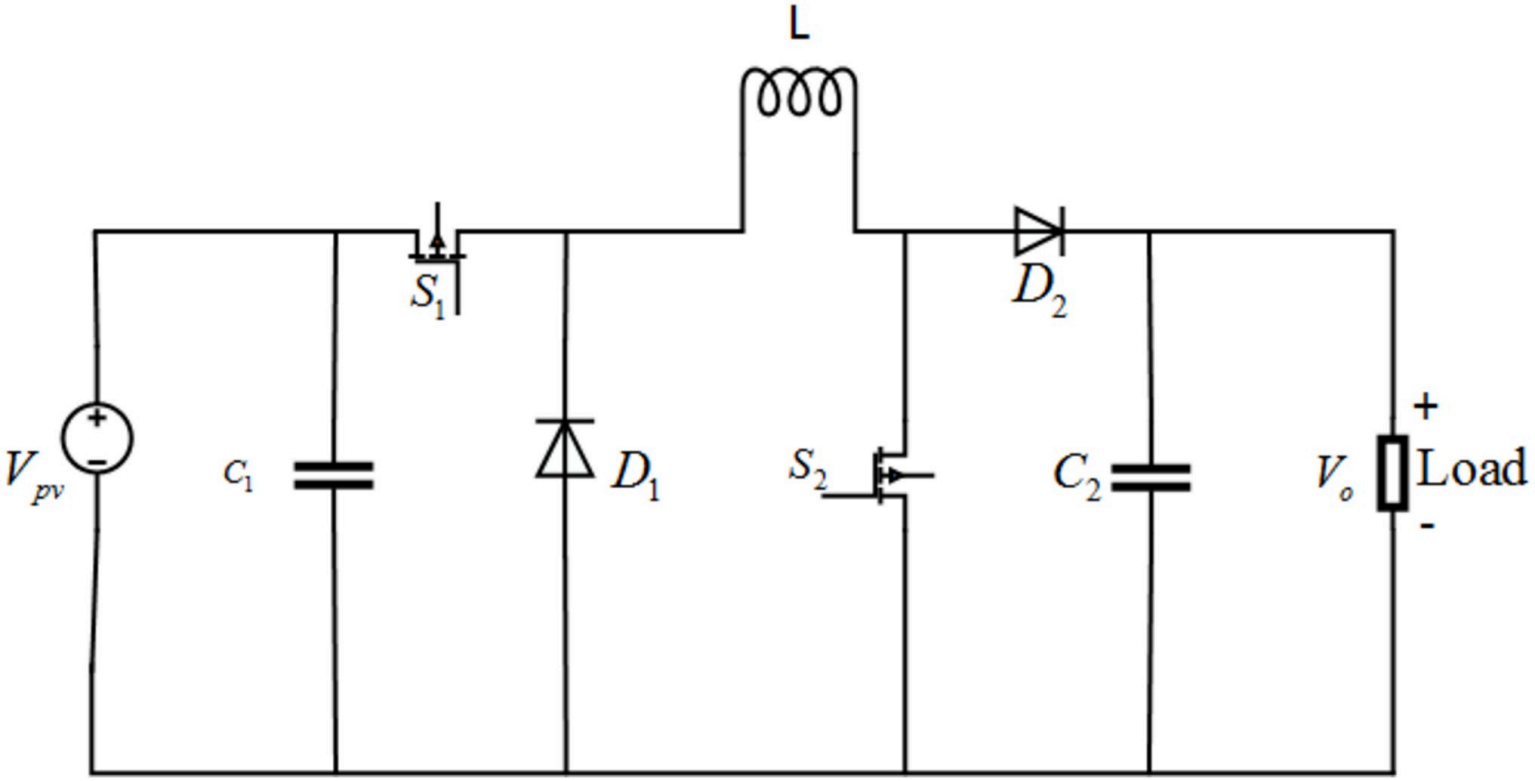
Circuit diagram of non-inverted buck boost converter.

In this work, a non-inverted Buck-Boost regulator is targeted [35]. It is a cascaded combination of buck converter followed by a boost converter. The converter is controlled periodically by the switching period, *T*_*s*_ whereas; *T*_*s*_ = *t*_*on*_ + *t*_*off*_. These converters are widely used in many applications such as industrial personal computers (IPCs), point-of-sale (POS) systems and auto start-stop systems [36]. The circuit arrangement of the non-inverted buck-boost converter is given in Fig 4. It is shown in the figure that *V*_*pv*_ is the input voltage from PV, *S*_1_ and *S*_2_ are insulated gate bipolar transistor IGBT switches, *L* represents the inductance of inductor, *D*_1_ and *D*_2_ are diodes, *C*_1_ and *C*_2_ are capacitance of the capacitors and *R* is the load resistance.

The expression for the inductor *L*, used in Fig 4, can be computed as follow [37]

$$\begin{array}{c} L=\frac{V_{pv}}{\Delta I_{L}f_{s}}D \end{array}$$ (10)

where Δ*I*_*L*_ = *I*_*max*_ − *I*_*min*_, *D* is the duty cycle and *f*_*s*_ is the switching frequency.

Similarly, the final expression for the output capacitor *C*_2_ carry the form [37]

$$\begin{array}{c} C_{2}=\frac{I_{C_{2}}}{\Delta V_{C_{2}}f_{s}}D \end{array}$$ (11)

where $\Delta V_{C_{2}}$ = $V_{c_{2,max}}-V_{c_{2,min}}$, *D* is the duty cycle and *f*_*s*_ is the switching frequency.

The detailed dynamics of the non-inverted buck-boost converter will be studied next.

### 3.1 Operating Modes of the buck-boost converter

Before proceeding to the mathematical modeling, the following assumption is made.

**Assumption 1** *Assume that the converter is operated in continuous conduction mode*.

The converter has two operating modes, i.e., Switch ON mode and Switch OFF mode, which is discussed below:

#### 3.1.1 Switch ON mode

**Fig 5:**
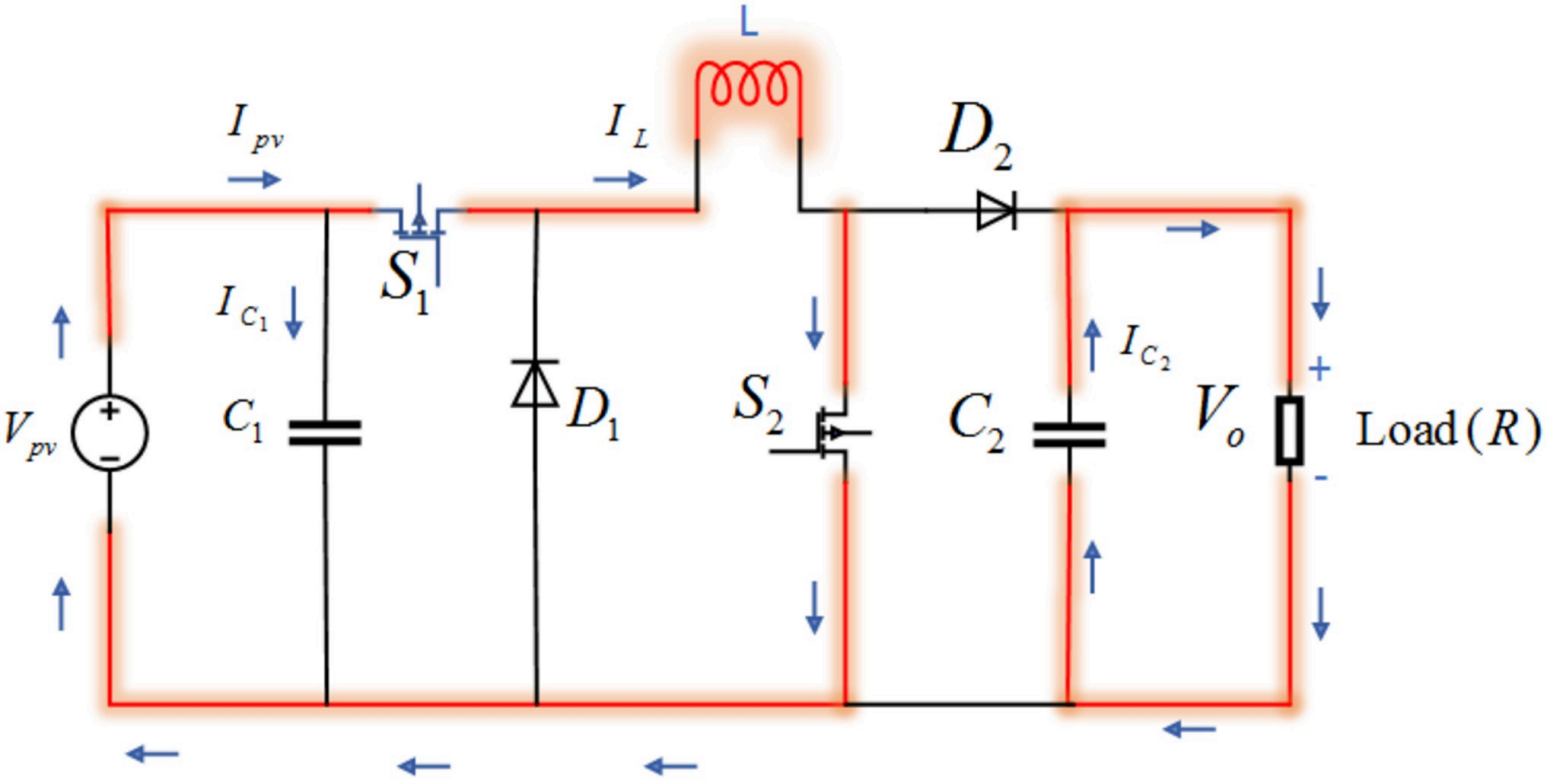
Switch ON mode of the non-inverted buck-boost converter.

In Switch ON mode, both the IGBT switches *S*_1_ and *S*_2_ are ON, which results in the disconnection of the load. So, the circuit on the left-hand side is shorted and the inductor charges from the PV voltage. The schematic diagram of the Switch ON mode is shown in Fig 5. By applying Kirchhoff’s laws, the state-space equations for the Switch ON mode can be derived as follows.

$$\left[\begin{array}{c} {\dot{V}}_{pv}\\ {\dot{I}}_{L}\\ {\dot{V}}_{o} \end{array}]=[\begin{array}{ccc} \frac{1}{C_{1}} & 0 & 0\\ 0 & \frac{1}{L} & 0\\ 0 & 0 & -\frac{1}{RC_{2}} \end{array}][\begin{array}{c} I_{pv}\\ V_{pv}\\ V_{o} \end{array}]+[\begin{array}{c} -\frac{I_{L}}{C_{1}}\\ 0\\ 0 \end{array}\right]$$ (12)

#### 3.1.2 Switch OFF mode

**Fig 6:**
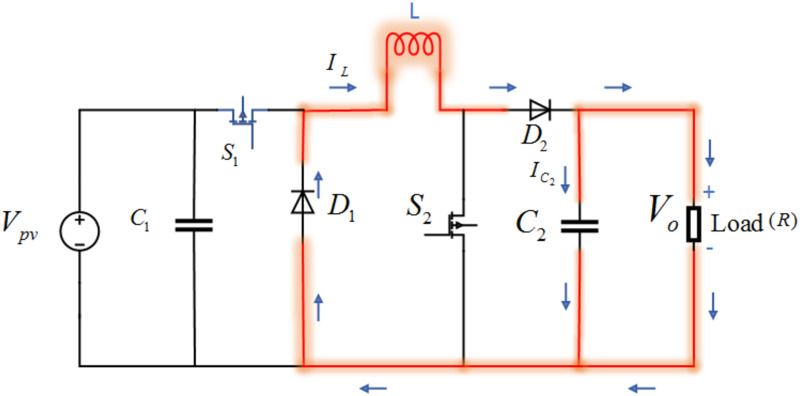
Circuit diagram of non-inverted buck boost converter.

In Switch-OFF mode, both the switches *S*_1_ and *S*_2_ are off. Now, in this case, the load is taking current from the inductor through diode *D*_2_ as shown in Fig 6.

The state-space equations for this mode by applying Kirchhoff’s laws can be obtained as

$$\left[\begin{array}{c} {\dot{V}}_{pv}\\ {\dot{I}}_{L}\\ {\dot{V}}_{C_{2}} \end{array}]=[\begin{array}{ccc} \frac{1}{C_{1}} & 0 & 0\\ 0 & \frac{1}{L} & 0\\ 0 & 0 & \frac{1}{C_{2}} \end{array}][\begin{array}{c} I_{pv}\\ -V_{o}\\ I_{L} \end{array}]+[\begin{array}{c} 0\\ 0\\ -\frac{V_{o}}{RC_{2}} \end{array}\right]$$ (13)

Based on the inductor volt-second balance and capacitor charge balance, the overall equations for both the modes of the non-inverted buck-boost converter are expressed as follow [38]

$$\left[\begin{array}{c} {\dot{V}}_{pv}\\ {\dot{I}}_{L}\\ {\dot{V}}_{C_{2}} \end{array}]=[\begin{array}{ccc} \frac{1}{C_{1}} & 0 & 0\\ 0 & \frac{D}{L} & \left(\frac{D}{L}-\frac{1}{L}\right)\\ 0 & 0 & -\frac{1}{RC_{2}} \end{array}][\begin{array}{c} I_{pv}\\ V_{pv}\\ V_{o} \end{array}]+[\begin{array}{c} -D\frac{I_{L}}{C_{1}}\\ 0\\ \left(\frac{I_{L}}{C_{2}}-D\frac{I_{L}}{C_{2}}\right) \end{array}\right]$$ (14)

Note that the duty cycle of a non-inverted buck-boost converter is used to control the output voltage, which is expressed as

$$\begin{array}{c} D=\frac{V_{o}}{V_{o}+V_{in}} \end{array}$$ (15)

In the case of ideal power transfer, the losses are ignored, so the input power is equal to output power. i.e. *P*_*i*_ = *P*_*o*_ and the input impedance *R*_*in*_ and output impedance *R* are related via the following equation.

$$\begin{array}{c} R_{in}=(\frac{1-D}{D}{)}^{2}R \end{array}$$ (16)

To design our control strategy, the model is averaged over one switching period. Consequently, the average value of *V*_*pv*_, *I*_*L*_ and *V*_*o*_ is considered as *x*_1_, *x*_2_, and *x*_3_, respectively, and *μ* is considered as the average duty cycle. Thus,

$$\begin{array}{c} \left[\begin{array}{cccc} x_{1} & x_{2} & x_{3} & \mu \end{array}]=[\begin{array}{cccc} V_{pv} & I_{L} & V_{C_{2}} & D \end{array}\right] \end{array}$$

using the notations, one may get the final state-space form as follows

$$\left[\begin{array}{c} {\dot{x}}_{1}\\ {\dot{x}}_{2}\\ {\dot{x}}_{3} \end{array}]=[\begin{array}{ccc} \frac{1}{C_{1}} & 0 & 0\\ 0 & \frac{\mu }{L} & \left(\frac{\mu }{L}-\frac{1}{L}\right)\\ 0 & 0 & -\frac{1}{RC_{2}} \end{array}][\begin{array}{c} I_{pv}\\ x_{1}\\ x_{3} \end{array}]+[\begin{array}{c} -\mu \frac{x_{2}}{C_{1}}\\ 0\\ \left(\frac{x_{2}}{C_{2}}-\mu \frac{x_{2}}{C_{2}}\right) \end{array}\right]$$ (17)

This system can also be written in a very compact form as follows

$$\begin{array}{c} \dot{x}=F\left(x,u\right) \end{array}$$

and

$$\begin{array}{c} y=x_{1} \end{array}$$

as the output voltage. In the subsequent section, the control design strategy is presented.

## 4 Reference voltage generation and design of the controller

This section presents the nonlinear controller design, which will be used in feedback mode to track an FFNN based generated reference signal (*V*_*ref*_). Therefore, at first, the reference signal generation is presented and then the control law designed is outlined.

### 4.1 FFNN based estimation of reference voltage *V*_*ref*_

In order to estimate the nonlinear mapping between the independent variables, i.e., temperature and irradiance, and the depending variable *V*_*ref*_ an FFNN [39], will be used. The neural networks (NN) learn to create a mapping between these independent variables and dependent output [40]. The training set in NN for this nonlinear approximation will include the temperature, variable irradiance and the reference voltage.

**Fig 7:**
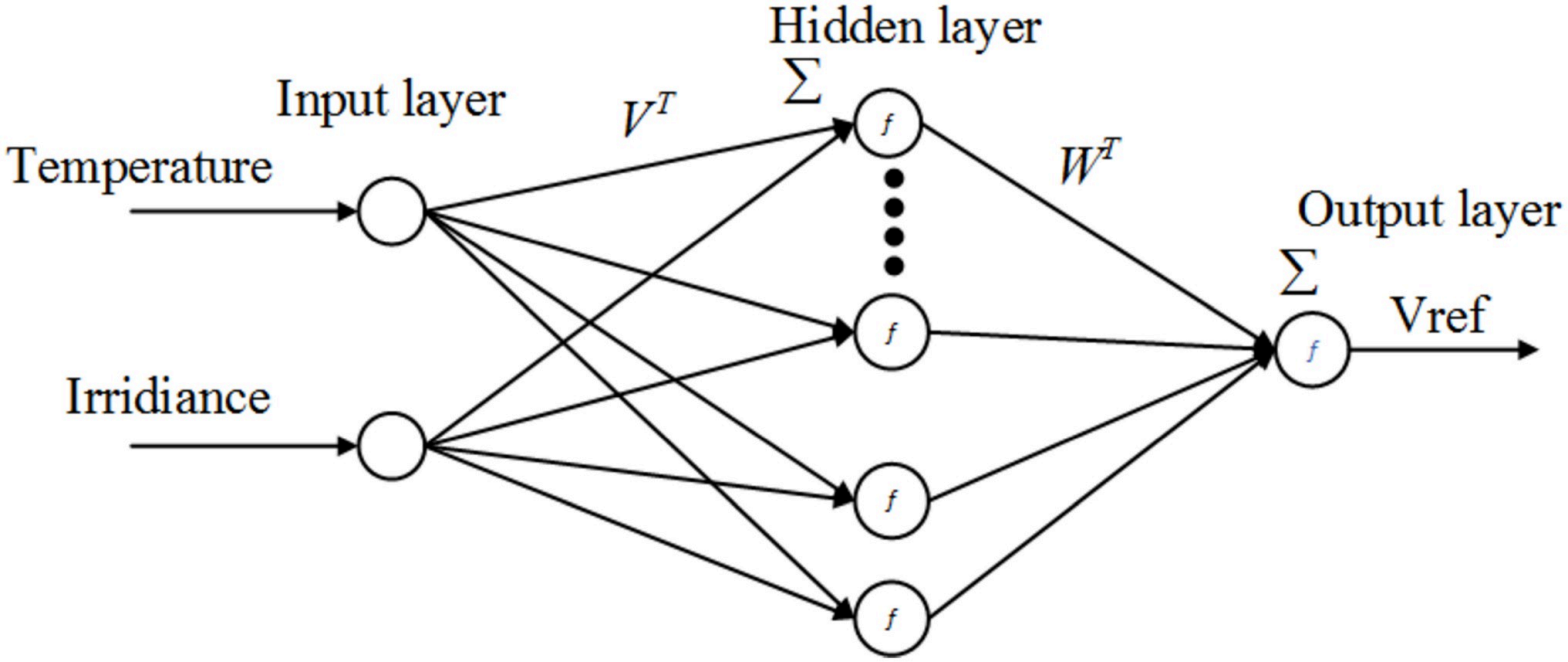
Neural network for *V*_*ref*_ generation.

Two-layer FFNN is efficient for any particular nonlinear estimation. Thus the proposed scheme with the schematic diagram, shown in Fig 7, is used for the estimation of the reference voltage (*V*_*ref*_), which will be tracked via the GGSMC to get MPP under varying irradiance and temperature.

The temperature (one of the network inputs) is varied from 25*C*° to 75*C*° with an increment of 2*C*°. In contrast, the irradiance (second input of the network) is varied from (600 − 1000)*w*/*m*^2^ with an appropriate increment of 1*w*/*m*^2^. The information in the input layer is multiplied by scalar weighted, *V*_*ij*_ with a biased value *b*_*jo*_. This input layer computes its net activation as

$$\begin{array}{c} a_{j}=\sum_{i=1}^{n}V_{ji}p_{i}+b_{jo} \end{array}$$ (18)

where *j* = 1, 2, 3.…, *l*_*o*_ represents the number of neurons in the hidden layer, *p*_*i*_ is the input of the input node *i*, *b*_*jo*_ is the respective reconstruction error or bias. The output of the hidden layer is given by

$$\begin{array}{c} y_{i}=f\left(a_{j}\right) \end{array}$$ (19)

where *f* is the activation function which is chosen to be tanh. The output layer computes its net activation as

$$\begin{array}{c} a_{k}=\sum_{j=1}^{l_{o}}\omega_{kj}y_{i}+b_{ko}, \end{array}$$ (20)

where *k* = 1 is the number of neurons in the output layer, *ω*_*kj*_ is scalar called weight between the *k*^*th*^ output layer node and the *j*^*th*^ hidden layer node. The output layer produces an output *V*_*ref*_ as a function of its net activation as

$$\begin{array}{c} V_{ref}=f\left(a_{k}\right) \end{array}$$ (21)

The output of the estimated model can be expressed as the function of inputs, the weights between input and hidden layer and the weights between the hidden and the output layer as described by

$$\begin{array}{c} V_{ref}=f(\sum_{j=1}^{l_{o}}\omega_{kj}f(\sum_{i=1}^{n}V_{ji}p_{i}+b_{jo})+b_{k0}) \end{array}$$ (22)

For two-layer FFNN, as shown in Fig 7, consider *l*_*o*_, and *k*_1_ are the number of neurons in layer1 and layer2, respectively. So the above equations can also be presented in the vectors form as

$$\begin{array}{c} V_{ref}=\bar{f}\left({\bar{W}}^{T}\bar{f}\left({\bar{V}}^{T}\bar{p}+b_{v}\right)+b_{w}\right) \end{array}$$ (23)

More explicitly, this expression can be expressed as follows.

$$\begin{array}{c} V_{ref}=\left(W\;\text{tanh}\;\left(V\bar{p}+b_{v}\right)+b_{w}\right) \end{array}$$ (24)

After selection of the network structure, the network training is done by minimizing the cost function, which is a function of network weights. The cost function is generally characterized as follows

$$\begin{array}{c} J\left(V_{ji},\omega_{kj}\right)=\frac{1}{2}\sum_{i=1}^{l_{o}}{\left(t_{k}-z_{k}\right)}^{2} \end{array}$$ (25)

where *t*_*k*_ is the target output at the *k*^*th*^ output node and *J*(*V*_*ji*_, *ω*_*kj*_) is the mean square error (MSE).

The levenberg-Marquardt training algorithm is used for updating the weights of the FFNN. The MSE criterion or the maximum number of iterations decides the termination of the iterative process. A range of values of the network parameters has been varied systematically to achieve a reasonable estimate of the training data. The varying network parameters are the number of hidden neurons in the hidden layers, with a learning rate range and the number of iterations is used for the estimation of *V*_*ref*_.

The final FFNN structure for *V*_*ref*_ has ten hidden layer neurons and the learning rate is 0.1. This choice of the network parameters yields a good match between the actual and the predicted values.

#### 4.1.1 Simulation results of FFNN

**Fig 8:**
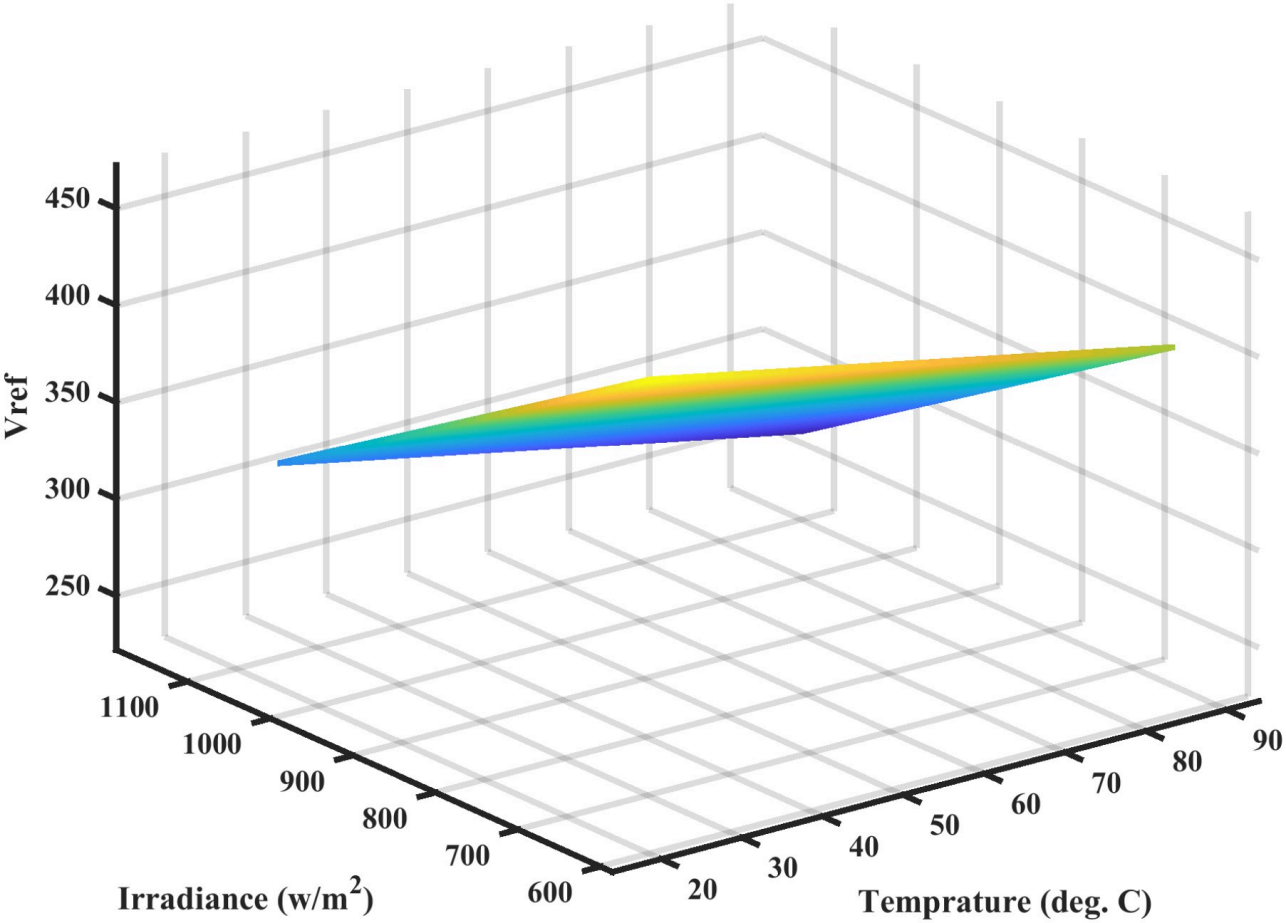
*V*_*ref*_ for different values of irradiance and temperature.

**Fig 9:**
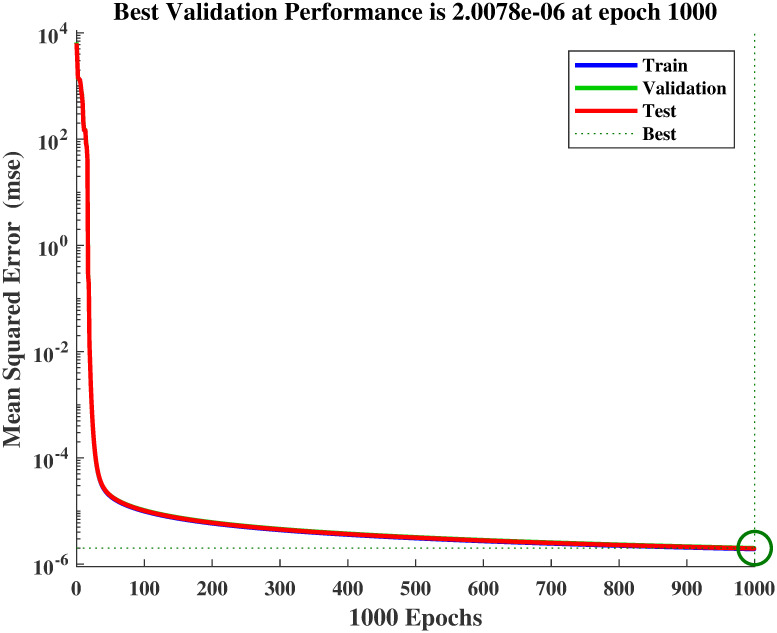
Mean square error convergence during the NN estimation.

**Fig 10:**
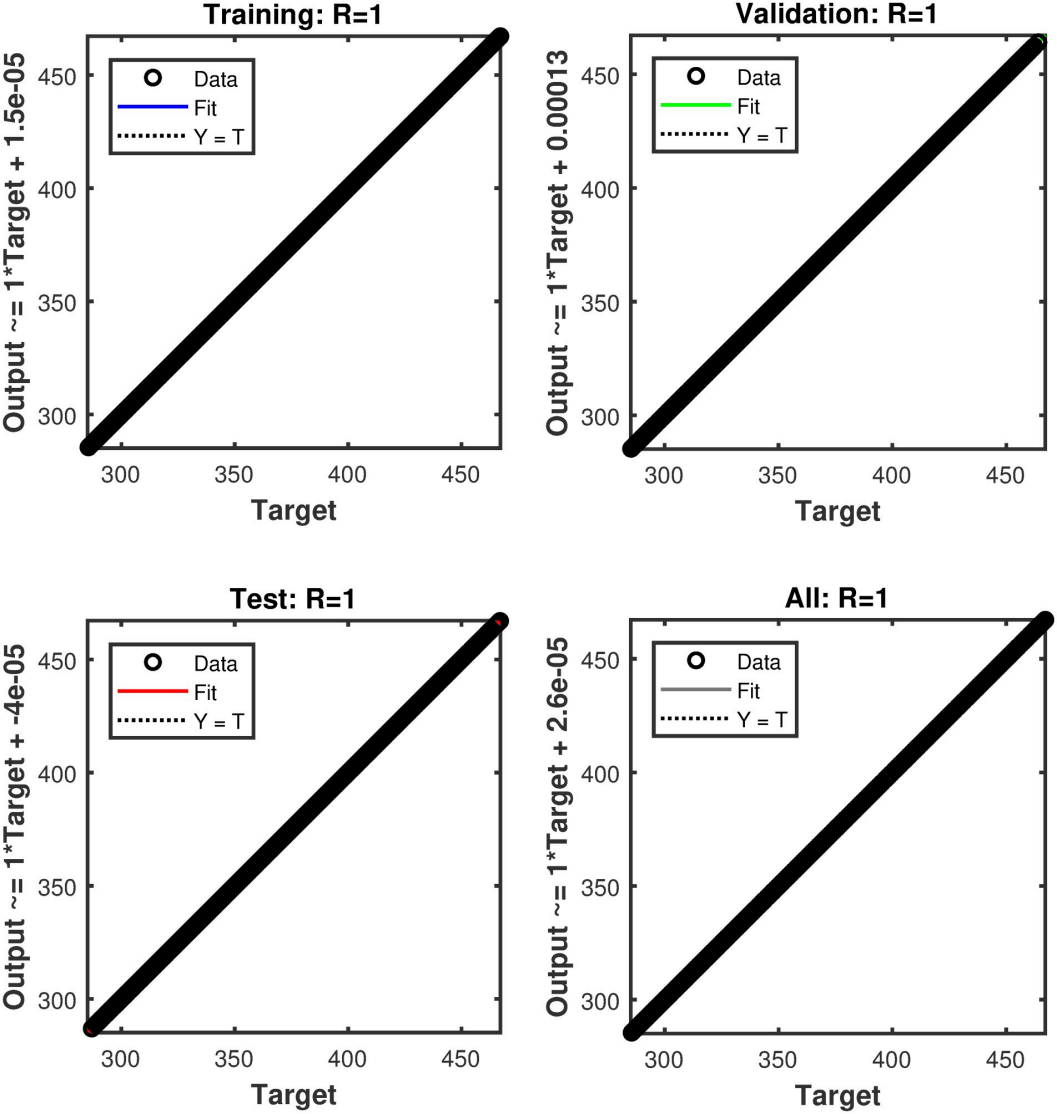
The regression plots during the estimation of *V*_*ref*_.

**Fig 11:**
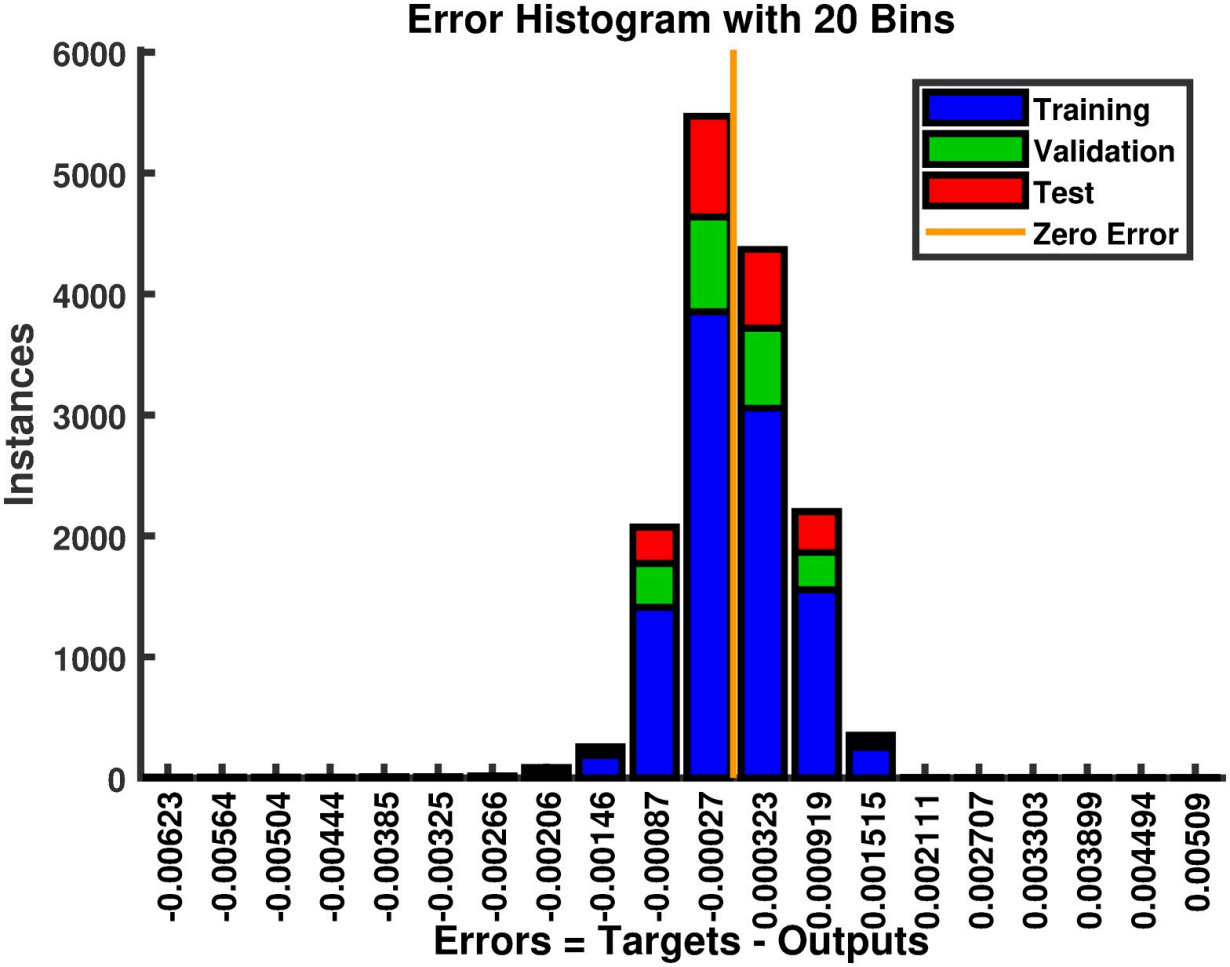
The error histogram during the estimation of *V*_*ref*_.

The reference voltage is *V*_*ref*_ generated for varying levels of temperature and irridiance as shown in Fig 8. The performance in terms of MSE, during the estimation of *V*_*ref*_, is shown in Fig 9. The regression plot of *V*_*ref*_ is shown in Fig 10. The estimates of *V*_*ref*_ are plotted against their target values in this figure. The regression value of *R* = 1 shows that the estimates are very close to the target values, indicating the success of FFNN training. The estimation error histogram associated with *V*_*ref*_ is shown in Fig 11. It reveals a very small error with an average very close to zero.

By providing *V*_*ref*_ by the FNN, the GGSM controller is then designed to steer *V*_*pv*_ to *V*_*ref*_ to get maximum power from the PV array.

### 4.2 Generalized global sliding mode control

To get maximum power from the PV module, a nonlinear GGSMC is designed to steer *V*_*pv*_ to *V*_*ref*_ by varying the duty cycle, i.e., *μ*

Before the control law design, the system (17) can be transformed into the following canonical form.

$$\begin{array}{c} \left\{ \begin{array}{c} \dot{y_{1}}=y_{2}\\ \dot{y_{2}}=\frac{1}{C_{1}}\left[{\dot{I}}_{pv}-\mu \dot{x_{2}}-x_{2}\dot{\mu }\right]+\Delta \left(y_{1},y_{2},t\right) \end{array}\right. \end{array}$$ (26)

where *y*_1_ = *x*_1_ and $y_{2}={\dot{x}}_{1}=\frac{I_{pv}}{C_{1}}-\mu \frac{x_{2}}{C_{1}}$ and Δ(*y*_1_, *y*_2_, *t*) is uncertainty which is assumed to be matched and bounded i.e. $|\Delta \left(y_{1},y_{2},t\right)|\leq \bar{K}$ with $\bar{K}$ is a positive constant.

This system (26) is a controllable canonical form in terms of the output and its derivative and is considered very convenient for the control design. The ultimate control objective is the tracking of the reference voltage *V*_*ref*_. The maximum power can be obtained by tracking this reference voltage. Therefore, the tracking error is the mismatch between the actual and reference voltages, i.e.,

$$\begin{array}{c} e=y-y_{ref} \end{array}$$

where *y*_*ref*_ = *V*_*ref*_. Based on this error, a new variable *σ* is defined as follows

$$\begin{array}{c} \sigma =\dot{e}+ae-f\left(t\right) \end{array}$$ (27)

Where the function *f*(*t*), called the forcing function, must be designed to meet the following three conditions so that *σ* = 0 [41].

$$\begin{array}{c} \left\{ \begin{array}{c} f\left(0\right)=\dot{e_{0}}+ae_{0}\\ f(t)\to 0\;\text{as}\;\text{t}\to \infty \\ f(t)\text{has}\;\text{a}\;\text{bounded}\;\text{first}\;\text{time}\;\text{derivative} \end{array}\right. \end{array}$$

Here $\dot{e_{0}}$ and *e*_0_ are the velocity and position errors respectively at *t* = 0. In GGSMC the function *f*(*t*) carry the following expression

$$\begin{array}{c} f\left(t\right)=\left[\left(\dot{e_{0}}+ae_{0}\right)\;\text{cos}\;bt-be_{0}\;\text{sin}\;bt\right]\text{exp}\left(-at\right) \end{array}$$ (28)

where *a* and *b* are positive constants. This forcing function satisfy all the above three conditions. The mathematical structure of (27) is made in such a way that it will result in the exponentially stable error convergence.

when *σ* = 0 then the solution of the closed loop system will be non homogeneous differential equation $\dot{e}+ae-f\left(t\right)=0$ will become

$$\begin{array}{c} e\left(t\right)=\left(e_{0}\;\text{cos}\;bt+\frac{\dot{e_{0}}+ae_{0}}{b}\text{sin}\;bt\right)\text{exp}\left(-at\right) \end{array}$$ (29)

This solution can also be obtained by solving the forthcoming second-order system.

$$\begin{array}{c} \ddot{e}+2a\dot{e}+\left(a^{2}+b^{2}\right)e=0 \end{array}$$ (30)

Where $\dot{e}\left(0\right)$ = ${\dot{e}}_{0}$ and *e*(0) = *e*_0_ are chosen as initial conditions.

This system displays that both the poles of the system are at −*a*±*bj*. This also indicates that the system’s dynamics in sliding mode do not reduce, which implies that the system experiences integral sliding mode.

The time derivative of (27) along system (26) becomes

$$\begin{array}{c} \begin{array}{c} \dot{\sigma }=\frac{1}{C_{1}}\left[{\dot{I}}_{pv}-\mu {\dot{x}}_{2}-x_{2}\dot{\mu }\right]-{\ddot{x}}_{ref}+a\left[\frac{I_{pv}}{C_{1}}-\mu \frac{x_{2}}{C_{1}}-{\dot{x}}_{ref}\right]-\dot{f}\left(t\right) \end{array} \end{array}$$ (31)

At this stage, the main interest is that the system should evolve with full states. Thus, a proportional-integral surface in term of *σ* can be defined as follows

$$\begin{array}{c} \xi =\sigma +c\int_{0}^{t}\sigma \left(\tau \right)d\tau \end{array}$$ (32)

The time derivative of *ξ* along (32) becomes

$$\begin{array}{c} \dot{\xi }=\dot{\sigma }+c\sigma \end{array}$$ (33)

Now putting the values of *σ*, $\dot{\sigma }$ and then posing $\dot{\xi }=0$.

The expression of the control law, which governs the system dynamics exactly on the manifold *ξ* = 0 can be calculated as follows

$$\begin{array}{c} \begin{array}{c} {\dot{u}}_{equ}=\frac{C_{1}}{x_{2}}\left[a\left\{\frac{I_{pv}}{C_{1}}-\mu \frac{x_{2}}{C_{1}}-{\dot{x}}_{ref}\right\}+\frac{1}{C_{1}}\left\{{\dot{I}}_{pv}-\mu {\dot{x}}_{2}\right\}-{\ddot{x}}_{ref}-\dot{f}\left(t\right)\right] \end{array} \end{array}$$ (34)

Since the practical system always operates under uncertainties, therefore, the equivalent control alone will be no more capable of enforcing sliding mode. So, the overall sliding mode enforcement law appear as follows

$$\begin{array}{c} \dot{u}={\dot{u}}_{equ}+{\dot{u}}_{dis} \end{array}$$ (35)

while

$$\begin{array}{c} u_{dis}=-k_{1}\left(\xi +Wsign\left(\xi \right)\right) \end{array}$$ (36)

**Fig 12:**
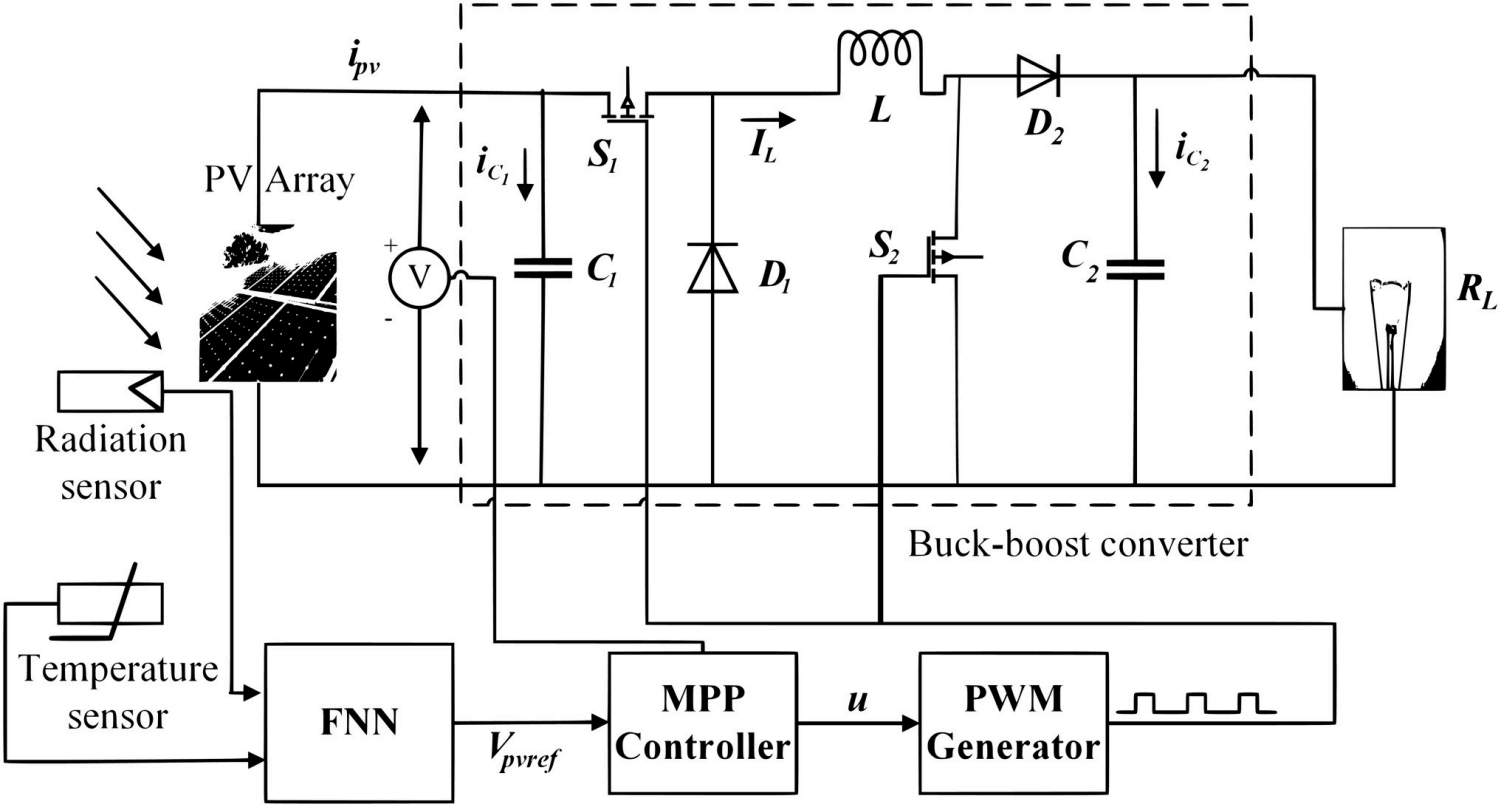
Proposed control scheme for PV power system.

Where 0 < *W* < 1. The schematic diagram of the designed control technique is shown in Fig 12.

### 4.3 Stability analysis

The close loop stability, i.e., the sliding mode enforcement and the actual states’ convergence to desired equilibrium points, can be confirmed by stating the following theorem.

**Theorem 1** *The sliding mode will take place along the manifold* (32) *by the control law of the* (35), *and hence the tracking will happen if the switching gain is chosen larger than the bound of the uncertainty i.e*.

$$\begin{array}{c} k_{1}>\left|\Delta \left(y_{1},y_{2},t\right)\right|+\eta \end{array}$$

*and, in addition, if the internal dynamics are asymptotically converging to equilibrium*.

**Proof 1** *We prove the theorem by first proving the asymptotic convergence of the internal dynamics since a two-step-based input-output form* (26) *was developed. This approach engages the first two dynamics equation of the system* (17); *thus, without loss of generality, the internal dynamics are governed by the last dynamic equation of* (17), *i.e*.,

$$\begin{array}{c} {\dot{x}}_{3}=-\frac{1}{RC_{2}}x_{3}+\frac{x_{2}}{C_{2}}-\frac{x_{2}}{C_{2}}\mu \end{array}$$ (37)

*Since x*_1_, *x*_2_ *are directly affected by the controlled input μ, so, the zero dynamics can be obtained by putting x*_1_, *x*_2_ *and μ* = 0, *one may get* (*see for details* [42]).

$$\begin{array}{c} {\dot{x}}_{3}=-\frac{1}{RC_{2}}x_{3} \end{array}$$ (38)

*Since, R and C*_2_ *are positive plant typical parameters, therefore*, $\frac{1}{RC_{2}}$ *will always remain positive. This confirms that the system* (38) *has poles in the left half-plane (LHP) at* $-\frac{1}{RC_{2}}$. *Hence* (38) *is the stable exponential system which implies that the system under study is the minimum phase. Now, at this stage, we are ready to confirm sliding mode establishment. A Lyapunov function, in terms of the sliding variable, is defined as follows*.

$$\begin{array}{c} V=\frac{1}{2}\xi^{2} \end{array}$$ (39)

*The time derivative of this function along* (34) *becomes*

$$\begin{array}{c} \dot{V}=\xi \dot{\xi }=\xi \left(\dot{\sigma }+c\sigma \right) \end{array}$$ (40)

*Using* (31) *and* (35) *(with components given in* (34) *and* (36), *one may get*

$$\begin{array}{c} \dot{V}=\xi \left(-k_{1}\left(\xi +Wsign\left(\xi \right)\right)+\Delta \right) \end{array}$$

$$\begin{array}{c} \dot{V}\leq -k_{1}\xi^{2}-k_{1}W|\xi |+\left|\xi ||\Delta \right| \end{array}$$ (41)

$$\begin{array}{c} \dot{V}\leq -k_{1}\xi^{2}-|\xi |(k_{1}W-\left|\Delta |\right) \end{array}$$

*This expression remains true, i.e., negative definite if*

$$\begin{array}{c} k_{1}W-\left|\Delta \right|\geq \eta >0 \end{array}$$

$$\begin{array}{c} k_{1}W\geq \eta +\left|\Delta \right| \end{array}$$ (42)

*using* (41) *and* (42), *one may get*

$$\begin{array}{c} \dot{V}\leq -k_{1}2V-\sqrt{2}\eta V^{\frac{1}{2}} \end{array}$$ (43)

$$\begin{array}{c} \dot{V}+k_{1}2V+\sqrt{2}\eta V^{\frac{1}{2}}\leq 0 \end{array}$$ (44)

*The differential inequality is a fast finite time converging, which forces V to zero and when V approaches zero, it means that ξ approaches zero. As ξ* → 0, (33) *will become*

$$\begin{array}{c} \dot{\sigma }+c\sigma =0 \end{array}$$ (45)

*The homogeneous differential* Eq (45) *has a solution*.

$$\begin{array}{c} \sigma (t)=\sigma (0)exp(-at) \end{array}$$ (46)

*It means that σ will approach zero. The σ convergence to zero certifies the* Eq (30) *with the solution reported in* (29). *Thus, the error converges to zero, which in turn provides us the reference tracking. Having tracked the reference, the maximum power will be extracted via the* Eq (16).

## 5 Simulation results

To verify the effectiveness and fast-tracking capability of the proposed controller, simulations are performed in MATLAB/Simulink R2018a environment under certain changes of environmental conditions. The typical parameters of the PV array and buck-boost converter specification used in the simulation are given in Table 1. The PV array has been connected to the load through a non-inverted buck-boost converter controlled by the FFNN based GGSMC. The simulation results are divided into two subsections. Firstly, the simulations are done with varying irradiance levels and, secondly, with varying temperature levels. The considered PV array in this simulation has 16 PV modules (Ns = 4, Np = 4), having 72 cells per module. The details of buck-boost converter specification and controller parameters detail are given in Table 2. Note that the trial and error method is used to select the values of controller parameters. In the process of parameters selection, we tried our best to choose and adjust these parameters on the premise of satisfying the designed conditions.

**Table 1:** PV array parameters and buck-boost converter specification.

Parameters	Values
Maximum power	1555W
Cells per module	72
Open circuit voltage	165.8 V
Short circuit current	17.56 A
*V*_*mpp*_	102.6 V
*I*_*mpp*_	15.16 A
Input capacitor *C*_1_	1 × 10^−3^ F
Output capacitor *C*_2_	48 × 10^−3^ F
Inductor *L*	20 × 10^−3^ H
Load *R*	50 Ohm

**Table 2:** Controller parameters.

Control parameters	Values
Constant *k*_1_	400
Constant *W*	0.0012
Constant *a*	500
Constant *b*	355

### 5.1 Test under varying irradiance

In this test, the temperature is kept constant at 25*C*° while varying the irradiance according to the following structure.

$$irr\left(t\right)=\{\begin{array}{cc} 650w/m^{2}, & \text{for}\;t\leq 0.2\;\text{sec}\\ 850w/m^{2}, & \text{for}\;0.2\;\text{sec}<t\leq 0.4\;\text{sec}\\ 1000w/m^{2}, & \text{for}\;t>0.4\;\text{sec} \end{array}$$

**Fig 13:**
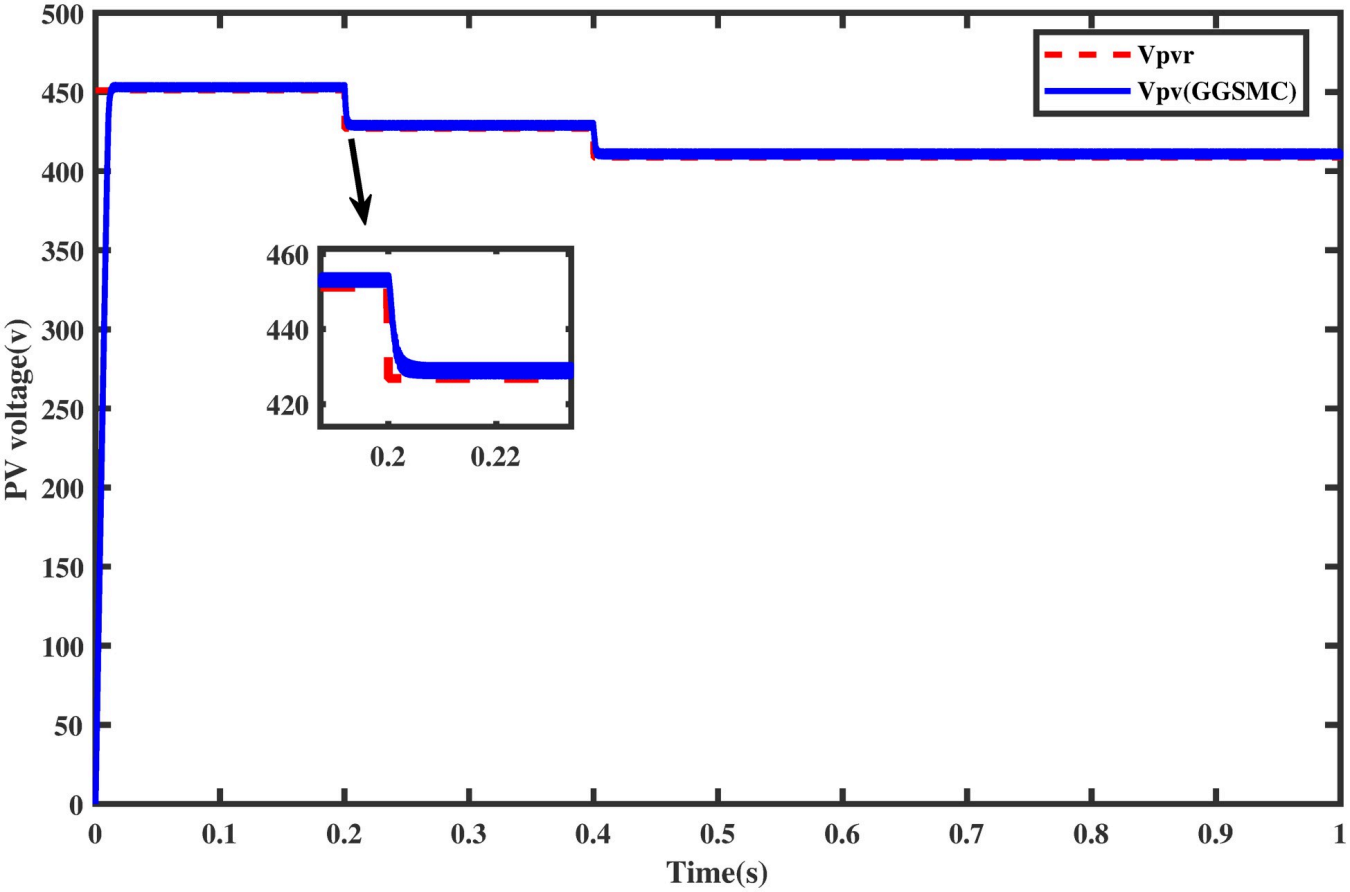
Tracking of *V*_*pv*_ with respect to *V*_*ref*_ under varying irradiance.

**Fig 14:**
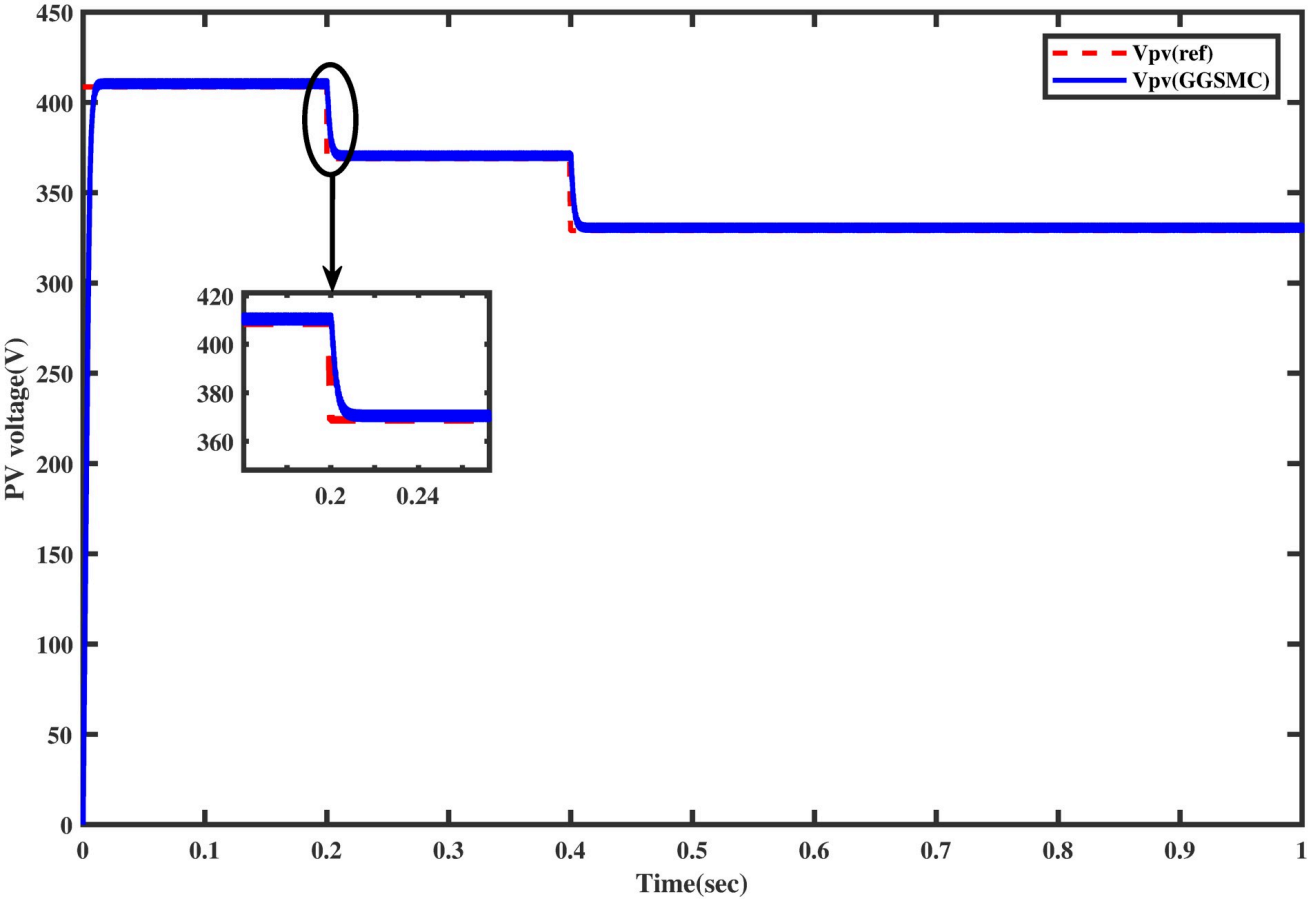
PV array power under varying irradiance.

The reference voltage *V*_*ref*_ is generated via FFNN while keeping in view the aforesaid temperature and irradiance profiles. The proposed controller tracks the reference voltage successfully, as shown in Fig 13. The corresponding output power of the PV array is also shown in Fig 14 which shows that MPP is achieved without oscillations. The performance of the newly proposed controller is relatively fast (without overshoots) during the variation in the irradiance profile. Thus, in this test, the proposed controller comes out to be a good candidate.

### 5.2 Test under varying temperature

For varying temperature, the irradiance is kept at 1000*w*/*m*^2^ and the temperature is varied as follows.

$$tem\left(t\right)=\{\begin{array}{cc} 25^{{}^{\circ}}, & \text{for}\;t\leq 0.2\;\text{sec}\\ 45^{{}^{\circ}}, & \text{for}\;0.2\;\text{sec}<t\leq 0.4\;\text{sec}\\ 65^{{}^{\circ}}, & \text{for}\;t>0.4\;\text{sec} \end{array}$$

**Fig 15:**
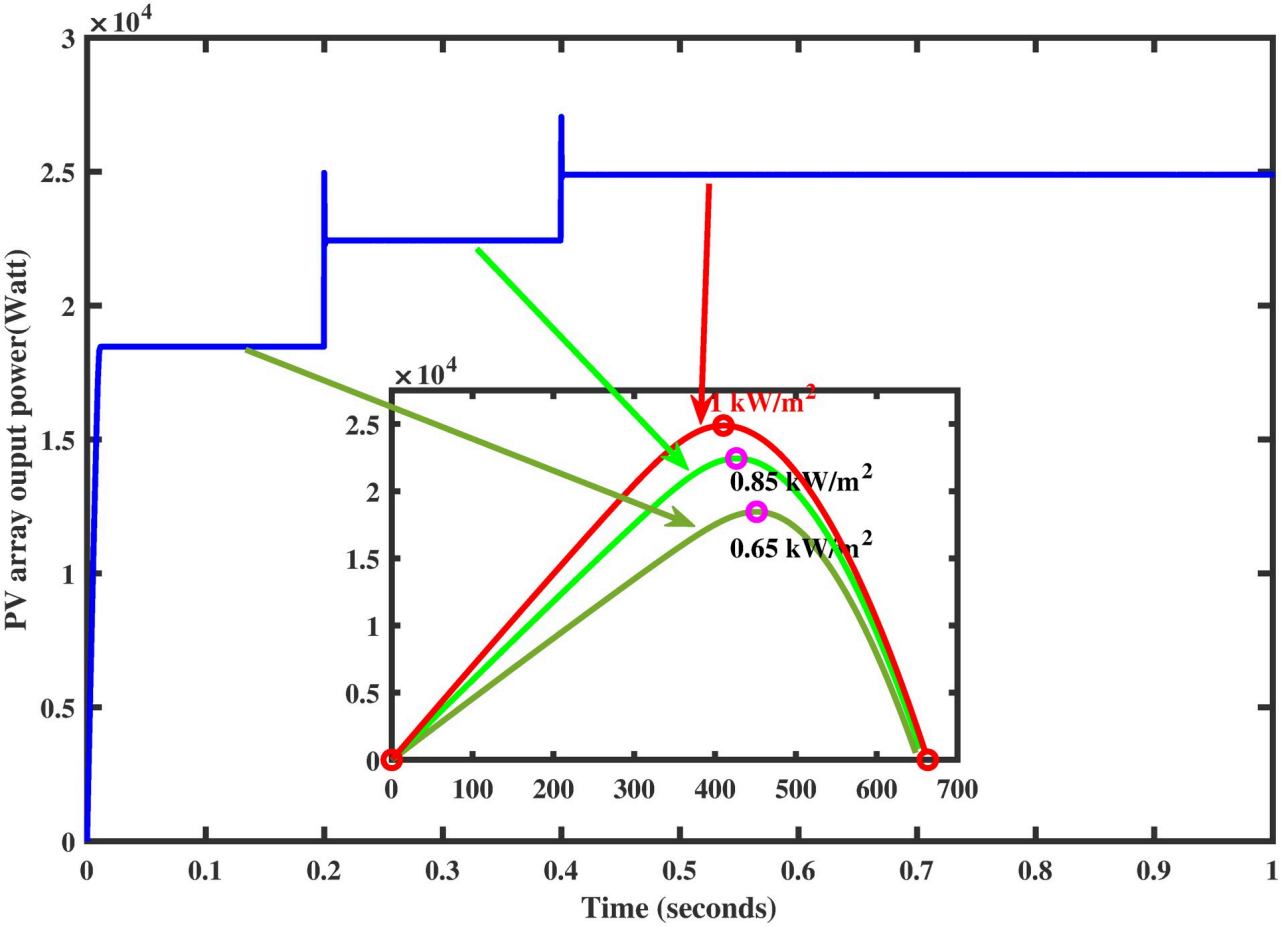
Tracking of *V*_*pv*_ with respect to *V*_*ref*_ under varying temperature.

**Fig 16:**
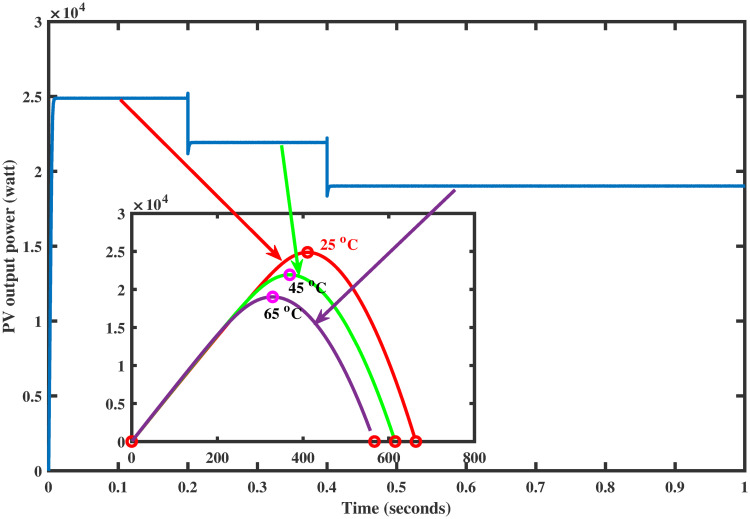
PV array power under varying temperature.

Again, the reference voltage, generated by FFNN for varying temperature levels, is tracked successfully via the proposed GGSM controller, as shown in Fig 15. The output power of the PV array is also shown in Fig 16 which shows that MPP is achieved without oscillations.

It is also shown that the proposed controller tracks the MPP during the abrupt variation in a short time of 0.003 sec.

### 5.3 Test under simultaneous variation in irradiance and temperature

**Fig 17:**
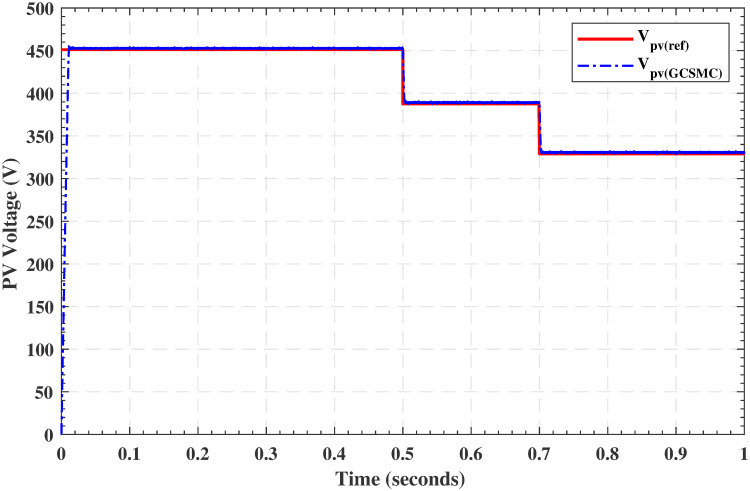
Tracking of *V*_*pv*_ with respect to *V*_*ref*_ under simultaneous variation in temperature and irradiance.

To show more effectiveness of the proposed work, the simulation results in Fig 17 investigate the MPPT performance of the proposed algorithm with simultaneous changes in irradiance and temperature. In this case, the temperature and irradiance are varied as follows

$$tem\left(t\right)=\{\begin{array}{cc} 25^{{}^{\circ}}, & \text{for}\;t\leq 0.5\;\text{sec}\\ 45^{{}^{\circ}}, & \text{for}\;0.5\;\text{sec}<t\leq 0.7\;\text{sec}\\ 65^{{}^{\circ}}, & \text{for}\;t>0.7\;\text{sec} \end{array}$$

$$irr\left(t\right)=\{\begin{array}{cc} 650w/m^{2}, & \text{for}\;t\leq 0.5\;\text{sec}\\ 846w/m^{2}, & \text{for}\;0.5\;\text{sec}<t\leq 0.7\;\text{sec}\\ 1000w/m^{2}, & \text{for}\;t>0.7\;\text{sec} \end{array}$$

From the above three tests, it is clear that the proposed controller provides maximum power in the case of varying irradiance and temperature. It is also evident that the transients are quite fast and acceptable. Hence, the proposed control law is a good and appealing algorithm for extracting maximum power in the variable scenario. The tracking performance of the FFNN based GGSMC is compared with the standard literature results in the subsequent subsection.

## 6 Comparison with standard literature results [38]

To effectively demonstrate the performance of the GGSMC, it is compared with backstepping controller [38]. Both the controllers are compared under varying temperature and irradiance levels, as shown below.

### 6.1 Comparison test under varying irridiance

**Fig 18:**
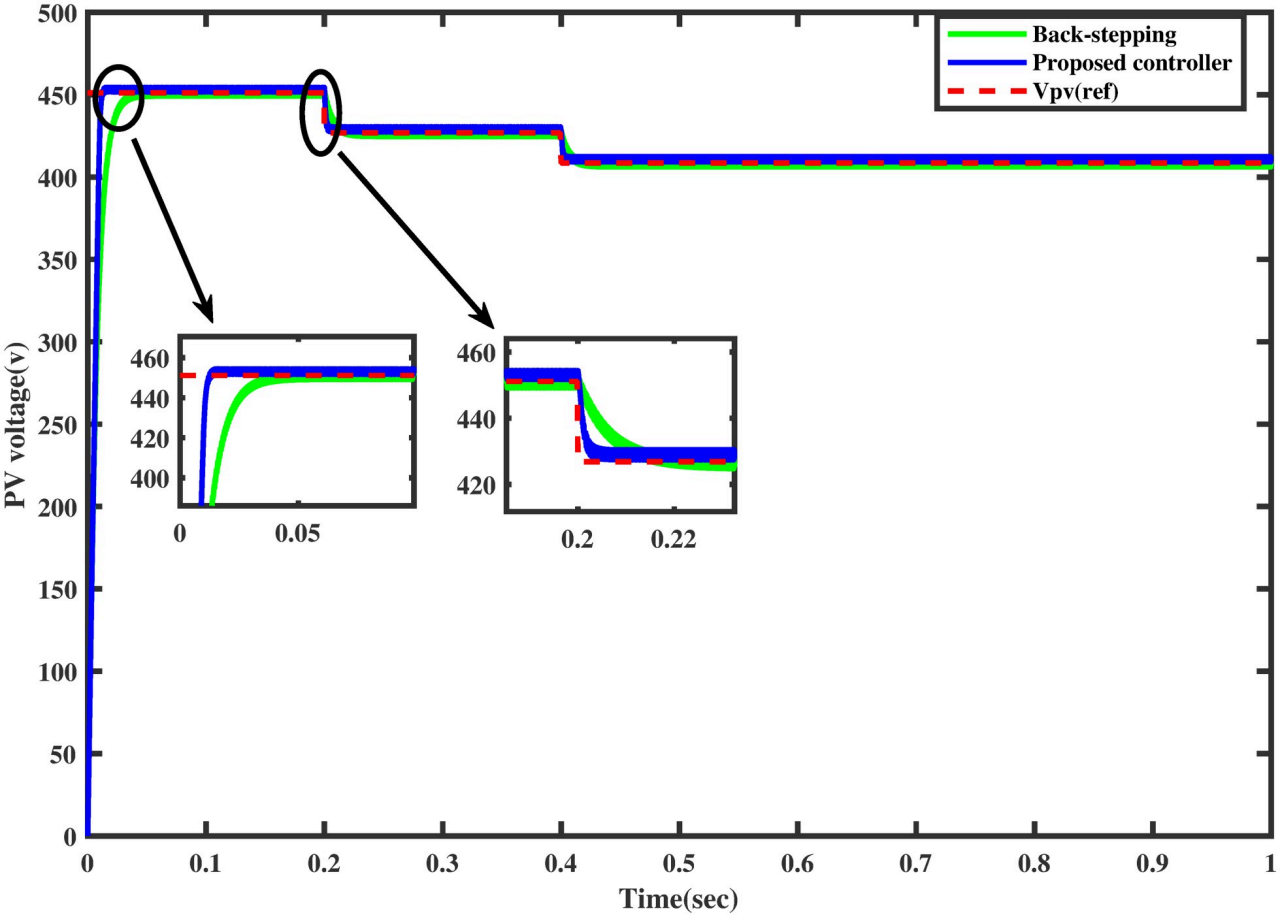
Comparison of PV array voltage under varying irradiance.

**Fig 19:**
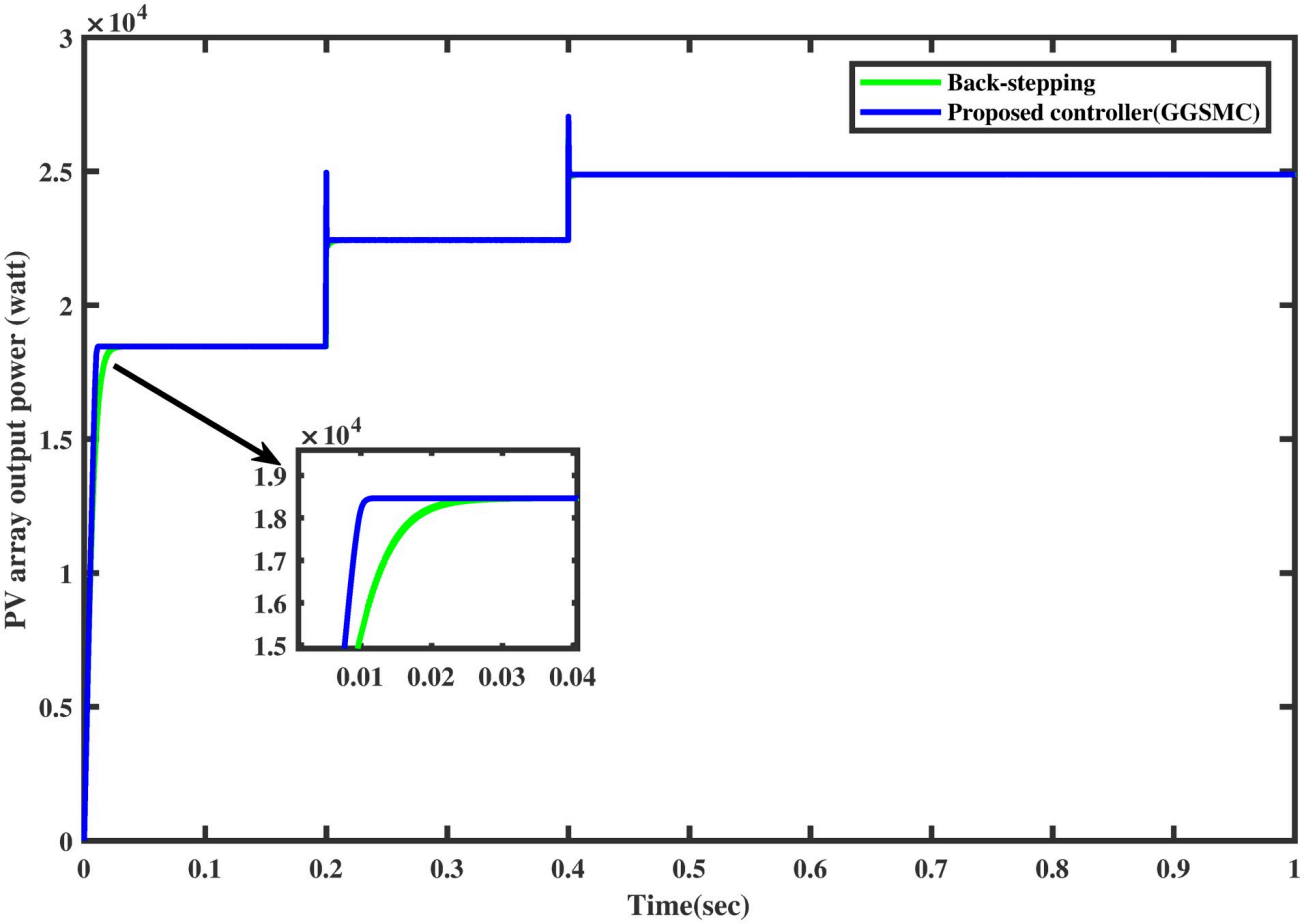
Comparison of PV array output power under varying irradiance.

For the same irradiance profile as in Section 6.1, the comparison of both the controllers is made as shown in Fig 18. It is seen clearly that the proposed controller reaches the MPP in a minimum time than the backstepping controller (one may see the zoomed view). Similarly, the proposed controller traces the MPP quickly when the irradiance level is stepped up after each 0.2 sec. The comparison of the output power of the PV array module with varying irradiance is displayed in Fig 19.

It is evident that the FFNN based GGSMC reaches the MPP rapidly, having low power losses as compared to backstepping controller [38].

### 6.2 Comparison test under varying temperature

**Fig 20:**
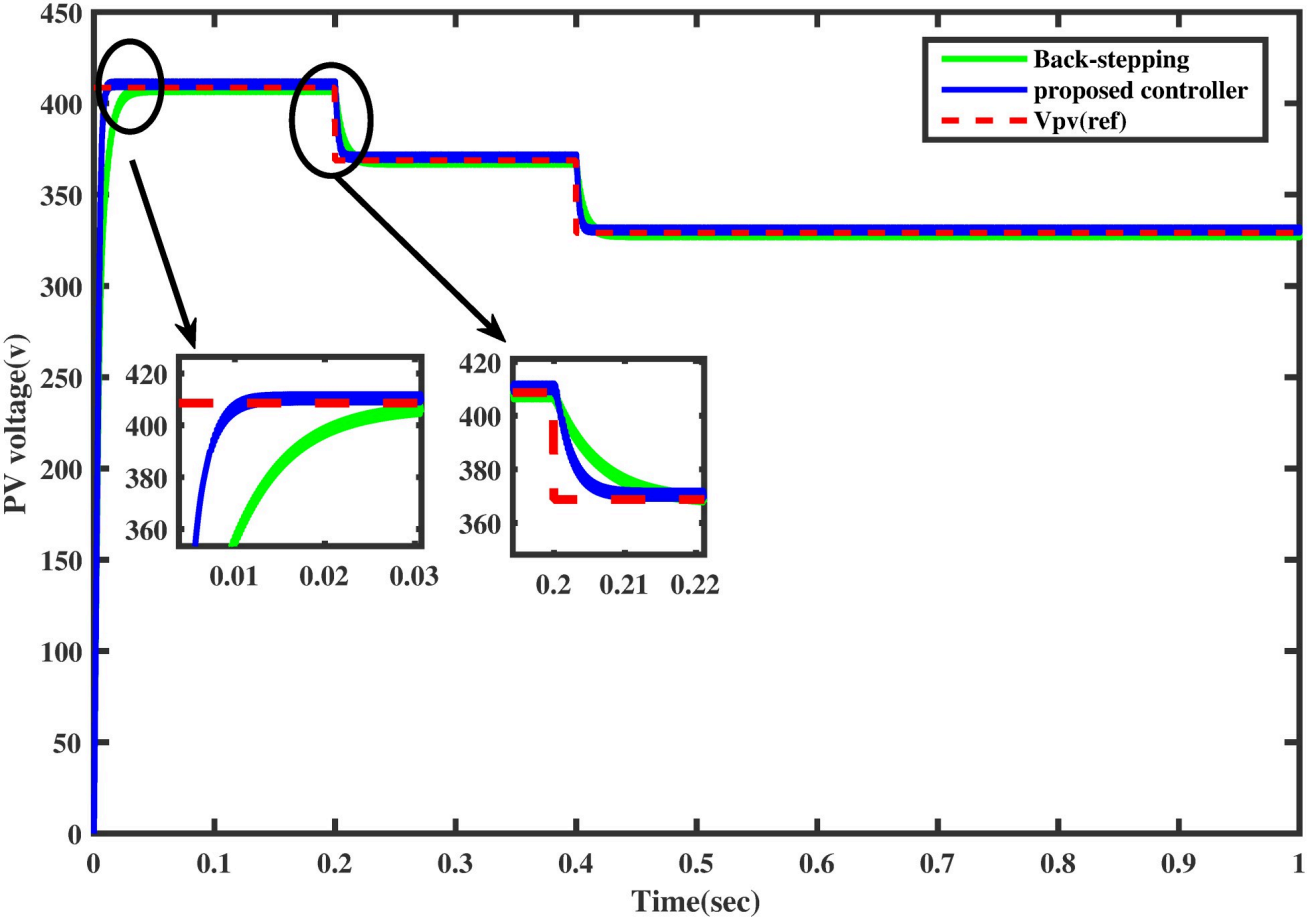
Comparison of PV array voltage under varying temperature.

**Fig 21:**
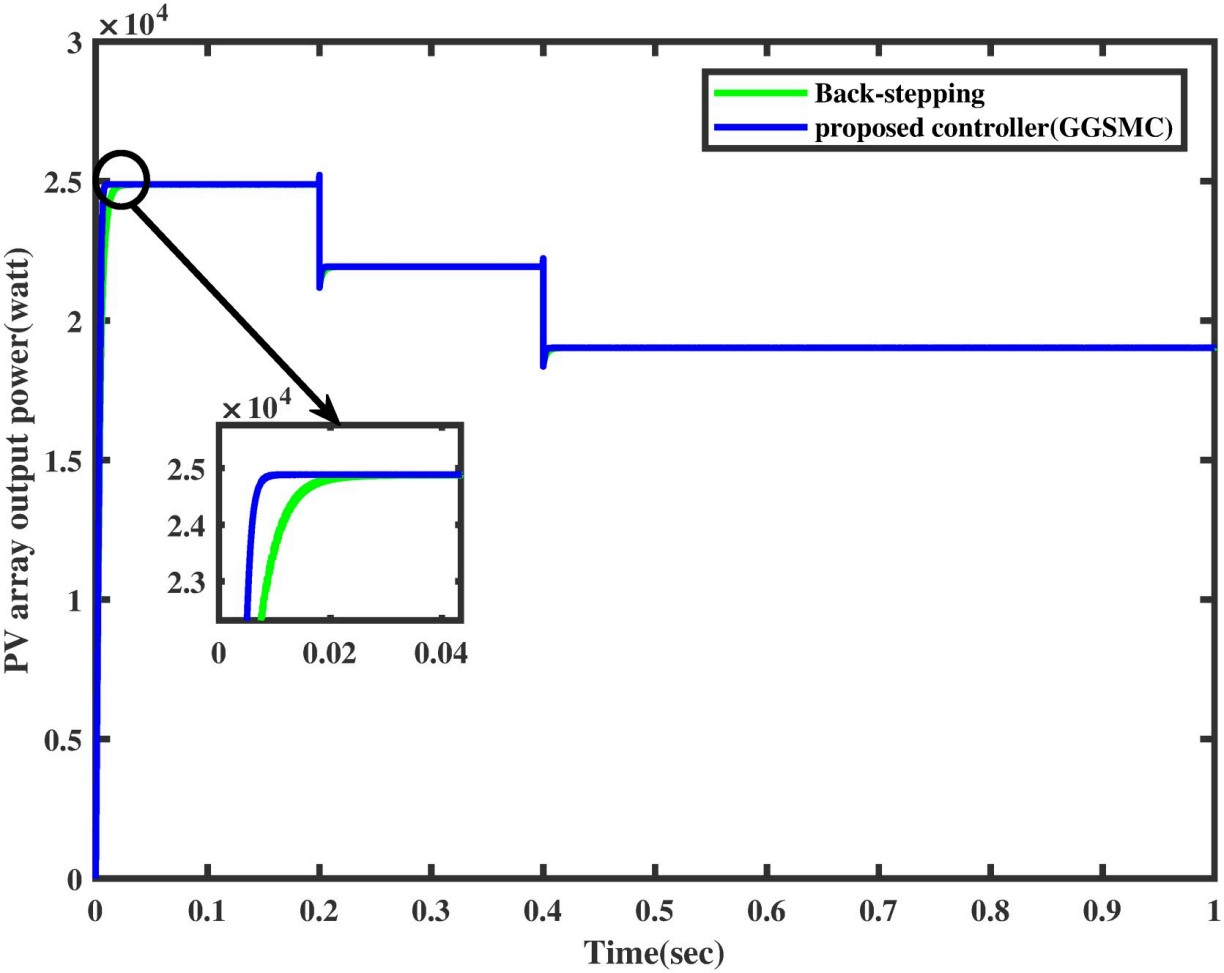
Comparison of PV array output power under varying temperature.

For the same temperature profile as in Section 5.2, this test is adapted for the comparison of both controllers. The comparative reference tracking is shown in Fig 20. It can be seen from the figure that the proposed controller reaches the MPP rapidly than the backstepping at the start and during the variation in temperature after each interval of 0.2 sec. In Fig 21, the comparison of the output power of the PV array module for both the controller shows that the proposed controller provides MPP rapidly with low power losses as compared to the backstepping controller.

### 6.3 Comparison test under simultaneous variation in temperature and irradiance

**Fig 22:**
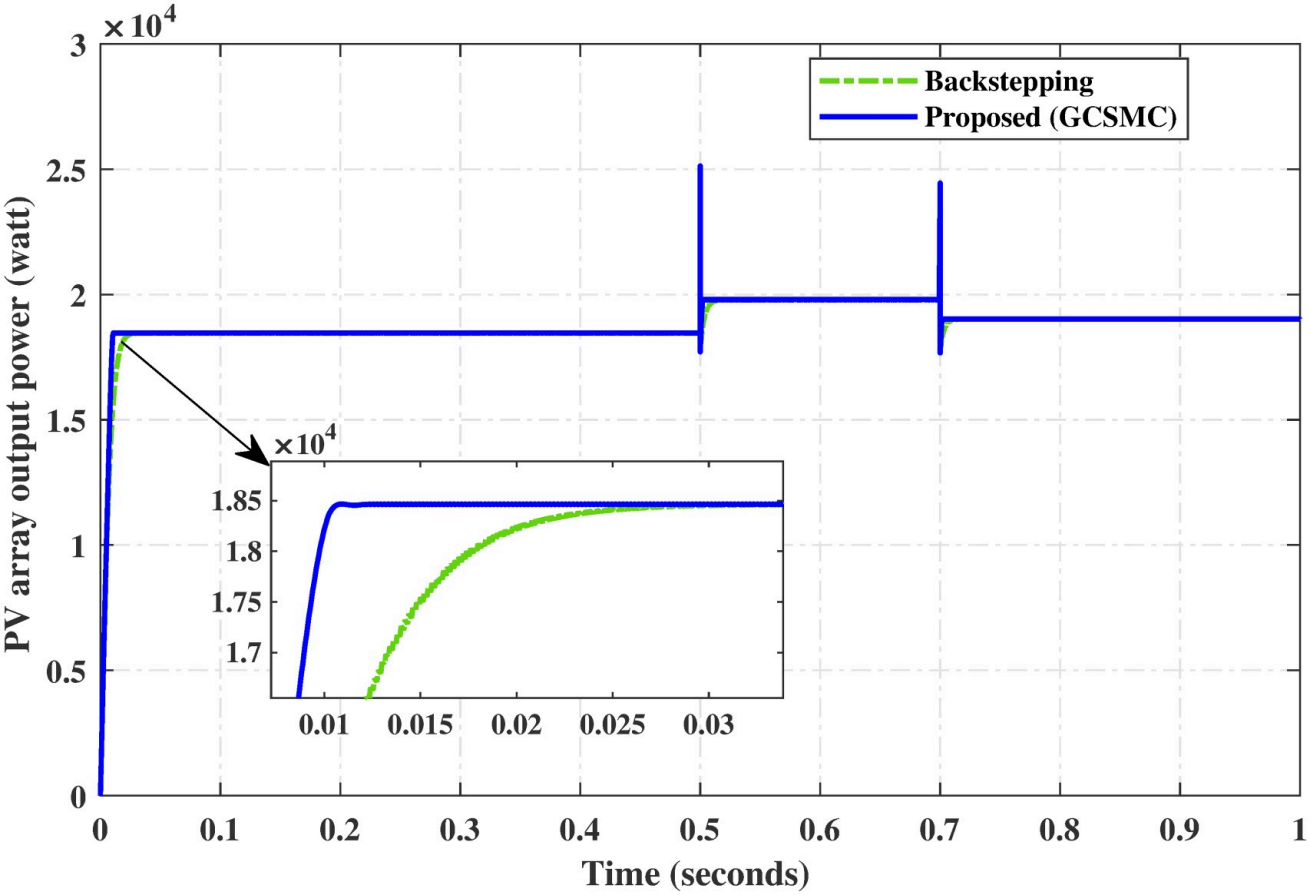
Comparison of PV array voltage under simultaneous variation in temperature and irradiance.

For varying profiles of temperature and irradiance (shown in Section 5.3), the simulation results of both controllers are compared. In Fig 22, the comparison of the output power of the PV array module for both the controller demonstrates that the proposed controller provides MPP rapidly with low power losses as compared to the reported backstepping controller.

## 7 Conclusion

Developing efficient and environment-friendly renewable energy systems is necessary to fulfill the energy demand and reduce environmental pollution. PV is one of such systems which generates energy under varying temperature and solar radiations. Therefore, in such varying scenarios, the maximum power extraction via a maximum power point tracking controller is very appealing and fascinating. In this work, a nonlinear GGSMC is derived from harvesting maximum power from a PV array with the help of a DC-DC buck-boost converter. For this purpose, FFNN is used to provide a reference voltage in the presence of varying environmental conditions. Having developed a reference voltage, a GGSMC is designed to track the generated reference voltage. The proposed control strategy eliminated the reaching phase, which resulted in enhanced robustness. The simulation results are developed in MATLAB/Simulink environment, demonstrating the proposed control’s effectiveness, accuracy, and rapid tracking. The results are compared with standard results of nonlinear backstepping controllers under abrupt changes in environmental conditions for further validation. Hence, it is concluded that our proposed control law demonstrates solid fast convergence with enhanced robustness with low power losses. In a nutshell, the FFNN based GGSMC is an appealing candidate for the PV systems operations. The future direction of this work includes GSMC based velocity observer design, adaptive control design against the system parametric variations, the practical implementation of the proposed controller on PV system, an efficient inverter design to inject harmonics free power of the PV system to the grid and its integration with the grid as well as other energy sources like fuel cell energy, biomass energy, etc.
